# Organelle communication maintains mitochondrial and endosomal homeostasis during podocyte lipotoxicity

**DOI:** 10.1172/jci.insight.182534

**Published:** 2024-08-08

**Authors:** Sho Hasegawa, Masaomi Nangaku, Yuto Takenaka, Chigusa Kitayama, Qi Li, Madina Saipidin, Yu Ah Hong, Jin Shang, Yusuke Hirabayashi, Naoto Kubota, Takashi Kadowaki, Reiko Inagi

**Affiliations:** 1 Division of Chronic Kidney Disease Pathophysiology and; 2 Division of Nephrology and Endocrinology, Graduate School of Medicine, The University of Tokyo, Tokyo, Japan.; 3 Department of Chemistry and Biotechnology, School of Engineering, The University of Tokyo, Tokyo, Japan.; 4 Department of Metabolic Medicine, Faculty of Life Science, Kumamoto University, Kumamoto, Japan.; 5 Department of Diabetes and Metabolic Diseases, and; 6 Department of Prevention of Diabetes and Lifestyle-Related Diseases, Graduate School of Medicine, The University of Tokyo, Tokyo, Japan.; 7 Toranomon Hospital, Tokyo, Japan.

**Keywords:** Nephrology, Chronic kidney disease, Mitochondria, Obesity

## Abstract

Organelle stress exacerbates podocyte injury, contributing to perturbed lipid metabolism. Simultaneous organelle stresses can occur in the kidney in the diseased state; therefore, a thorough analysis of organelle communication is crucial for understanding the progression of kidney diseases. Although organelles closely interact with one another at membrane contact sites, limited studies have explored their involvement in kidney homeostasis. The endoplasmic reticulum (ER) protein, PDZ domain–containing 8 (PDZD8), is implicated in multiple-organelle-tethering processes and cellular lipid homeostasis. In this study, we aimed to elucidate the role of organelle communication in podocyte injury using podocyte-specific *Pdzd8*-knockout mice. Our findings demonstrated that *Pdzd8* deletion exacerbated podocyte injury in an accelerated obesity–related kidney disease model. Proteomic analysis of isolated glomeruli revealed that *Pdzd8* deletion exacerbated mitochondrial and endosomal dysfunction during podocyte lipotoxicity. Additionally, electron microscopy revealed the accumulation of abnormal, fatty endosomes in *Pdzd8*-deficient podocytes during obesity-related kidney diseases. Lipidomic analysis indicated that glucosylceramide accumulated in *Pdzd8*-deficient podocytes, owing to accelerated production and decelerated degradation. Thus, the organelle-tethering factor, PDZD8, plays a crucial role in maintaining mitochondrial and endosomal homeostasis during podocyte lipotoxicity. Collectively, our findings highlight the importance of organelle communication at the 3-way junction among the ER, mitochondria, and endosomes in preserving podocyte homeostasis.

## Abstract 2

Organelle interactions at the three-way junction among the endoplasmic reticulum, mitochondria and endosomes are critical for preserving podocyte homeostasis during obesity-related kidney diseases.

## Graphical abstract

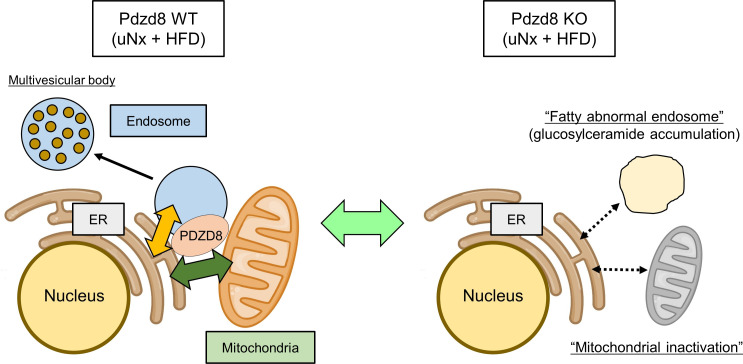

## Introduction

Organelles are confined functional subunits within the cell. Various essential biochemical reactions for maintaining cellular homeostasis, including energy metabolism, protein quality control, and degradation, occur within organelles. The cytoplasmic separation of organelles enables each to have a distinct role and perform complex cellular functions (1).

Organelle stress is a major exacerbating factor in podocyte injury, leading to dysregulated lipid metabolism. For example, mitochondrial dysregulation compromises mitochondrial lipid utilization, ultimately resulting in lipid accumulation and lipotoxicity (2). Additionally, in an obesity-related kidney disease model, podocyte-specific autophagy-deficient mice exhibit more severe albuminuria and podocyte damage than their littermate controls (3).

Recent advances in imaging techniques have revealed close interactions between organelles at membrane contact sites (MCSs). For example, mitochondria and the endoplasmic reticulum (ER) form mitochondria-ER contact sites (MERCSs), serving as a unique subcellular platform for metabolite exchange, such as Ca^2+^ and glycerophospholipids (4, 5). Furthermore, endosome maturation, involving the conversion of early endosomes to late endosomes for subsequent fusion with lysosomes, occurs at contact sites between endosomes and the ER through lipid transfer in neuronal cells (6, 7). Thus, organelle communication via MCSs is crucial for cellular homeostasis. However, few studies have explored the involvement of MCSs in kidney homeostasis, partly owing to technical challenges in analyzing in vivo organelle communication.

The ER protein PDZ domain–containing 8 (PDZD8) plays a pivotal role in multiple-organelle-tethering processes and cellular lipid homeostasis. PDZD8 is essential for MERCS formation, mediating Ca^2+^ transfer from the ER to mitochondria in various cell types (4, 8, 9). Additionally, PDZD8 is located at ER-endosome contact sites, promoting endosome maturation through lipid transfer (7, 8, 10, 11).

To address the gaps in the literature, in this study, we aimed to elucidate the involvement of organelle communication and perturbed lipid homeostasis in podocyte injury, with a specific focus on the function of PDZD8 as a multiple-organelle-tethering factor.

## Results

### Pdzd8 deletion induces podocyte endosomal malformation in an obesity-related kidney disease.

**Figure 1:**
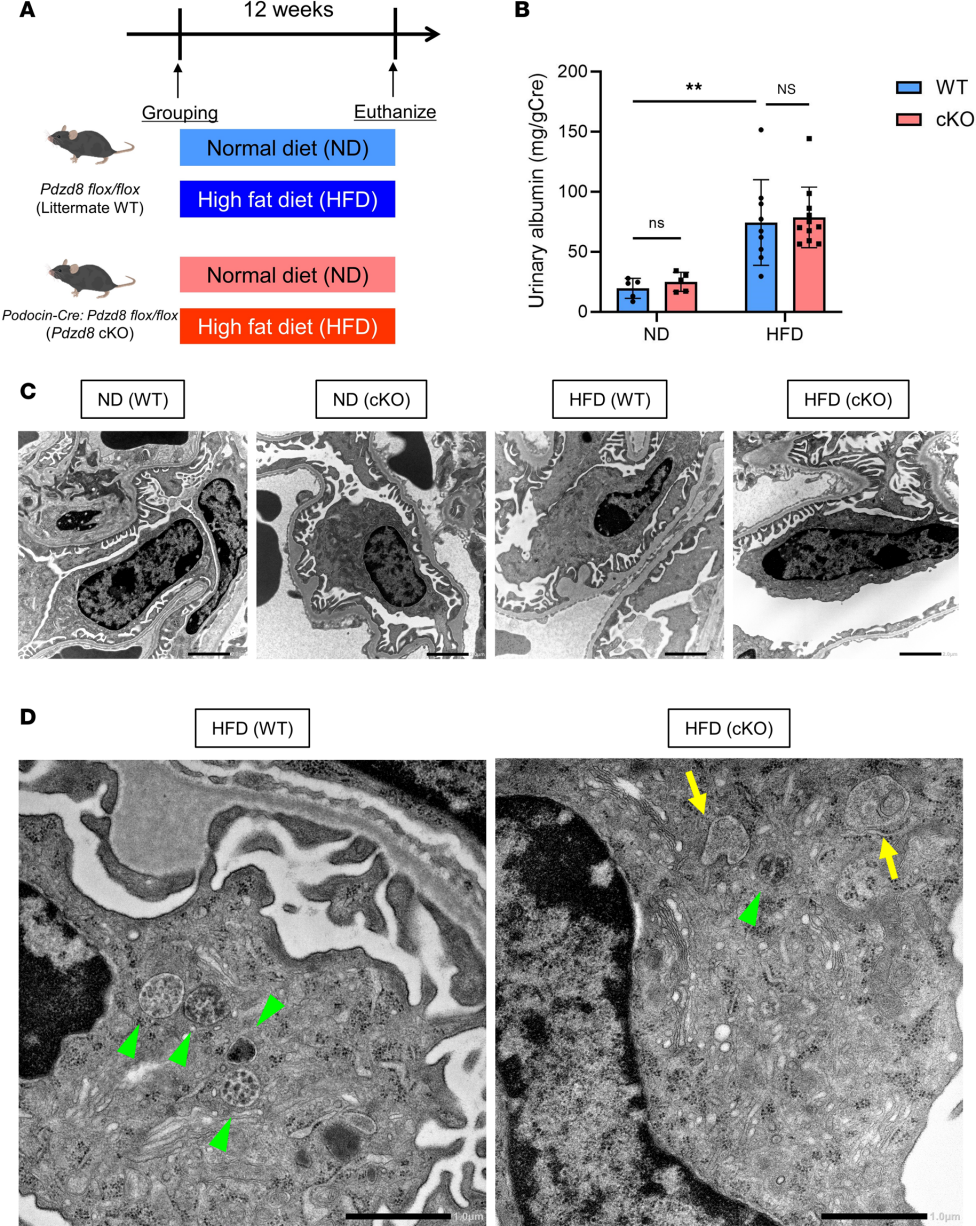
*Pdzd8* deletion induces podocyte endosomal malformation in an obesity-related kidney disease model. (**A**) The study design is shown (ND: *n* = 5, HFD: *n* = 9 or 11). (**B**) The urinary albumin levels are shown. (**C**) The electron microscope imaging with low magnification is shown. Scale bars: 2 μm. (**D**) The electron microscope imaging with high magnification is shown. Scale bars: 1 μm (green arrowheads: normal endosomes, yellow arrows: abnormal endosomes). WT, wild-type control mice; cKO, podocyte-specific *Pdzd8*-knockout mice. Data are presented as mean ± SD. *P* values were determined by 2-way ANOVA with Tukey’s multiple-comparison test. **P < 0.01. NS, not significant.

Podocyte-specific *Pdzd8* conditional knockout mice (podocin-Cre: *Pdzd8^fl/fl^*, cKO) and littermate wild-type controls (*Pdzd8^fl/fl^*, WT) were generated. Genotype analysis was conducted using tail PCR (Supplemental Figure 1, A and B; supplemental material available online with this article; https://doi.org/10.1172/jci.insight.182534DS1), and *Pdzd8* deletions in podocytes were validated using quantitative real-time PCR of the isolated glomeruli (Supplemental Figure 1C). The mice were divided into 2 groups and fed either a normal diet (ND) or a high-fat diet (HFD) for 12 weeks (Figure 1A).

Urinary albumin levels, a marker of podocyte injury, were higher in the HFD group than that in the ND group. However, urinary albumin levels did not significantly differ between the HFD-WT and HFD-cKO groups (Figure 1B). Background data, such as body weight, plasma cholesterol, plasma triglyceride, blood urea nitrogen (BUN), and plasma creatinine levels, did not significantly differ between the HFD-WT and HFD-cKO groups (Supplemental Figure 2, A–E). Although optical microscope imaging using periodic acid–Schiff (PAS) staining did not reveal significant differences between HFD-WT and HFD-cKO (Supplemental Figure 2F), we observed the presence of abnormal endosomes in HFD-cKO groups using electron microscopy (Figure 1, C and D; yellow arrows), resembling abnormal large vacuoles observed in *Pdzd8*-deleted HeLa cells and neurons in previous studies (7).

### Pdzd8 deletion affects mitochondrial and endosomal homeostasis during podocyte lipotoxicity.

**Figure 2:**
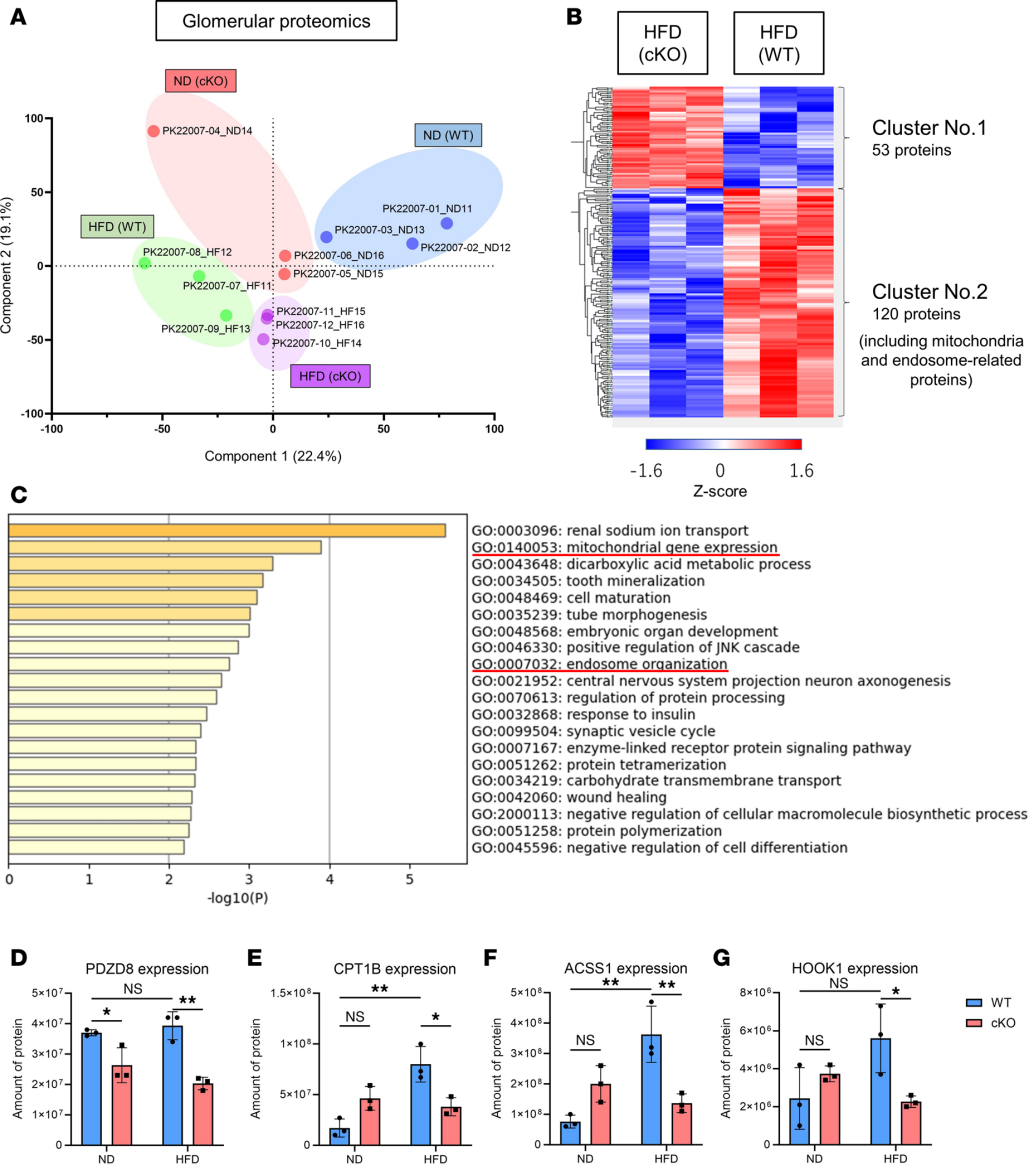
Glomerular proteomics data of an obesity-related kidney disease model using podocyte-specific *Pdzd8*-knockout mice. (**A**) Principal component analysis (PCA) plot of the comprehensive proteomics of isolated glomeruli is shown. (**B**) Heatmap of differentially expressed proteins between HFD-WT and HFD-cKO groups is shown. (**C**) Gene ontology (GO) analysis of 173 differentially expressed proteins between HFD-WT and HFD-cKO groups is shown. GO terms related to mitochondrial and endosomal homeostasis are underlined in red. (**D**) The amounts of PDZD8, (**E**) carnitine palmitoyltransferase 1b (CPT1B), (**F**) acyl-CoA synthetase short-chain family member 1 (ACSS1), and (**G**) hook microtubule–tethering protein 1 (HOOK1) are shown (*n* = 3, each). ND, normal diet; HFD, high-fat diet; WT, wild-type control mice; cKO, podocyte-specific *Pdzd8*-knockout mice. Data are presented as mean ± SD. *P* values were determined by 2-way ANOVA with Tukey’s multiple-comparison test. *P < 0.05, **P < 0.01. NS, not significant.

To elucidate the mechanism underlying the emergence of abnormal endosomes in the podocytes of HFD-cKO mice, we isolated glomeruli and performed a comprehensive proteomic analysis (Supplemental Table 1; jPOST repository accession number JPST002955). The ND-WT, ND-cKO, HFD-WT, and HFD-cKO groups were distinctly distributed in principal component analysis (PCA) plots, indicating differences in proteomic profiles (Figure 2A). Notably, mitochondrial gene expression and endosome organization were enriched by the gene ontology (GO) analysis of 173 differentially expressed proteins between the HFD-WT and HFD-cKO groups (Figure 2, B and C, and Supplemental Table 2). When examining individual proteins, PDZD8 levels were reduced in the cKO group compared with the WT group (Figure 2D). The quantity of proteins related to mitochondrial fatty acid oxidation (FAO), including carnitine palmitoyltransferase 1b (CPT1B) and acyl-CoA synthetase short-chain family member 1 (ACSS1), was lower in HFD-cKO mice than that in HFD-WT mice (Figure 2, E and F). Additionally, the level of hook microtubule–tethering protein 1 (HOOK1), which is related to endosome organization, was lower in HFD-cKO mice than that in HFD-WT mice (Figure 2G). Collectively, a comprehensive proteomic analysis of isolated glomeruli revealed that *Pdzd8* deletion affects mitochondrial and endosomal homeostasis during podocyte lipotoxicity.

### Pdzd8 deletion exacerbates podocyte injury in an accelerated obesity–related kidney disease model.

**Figure 3:**
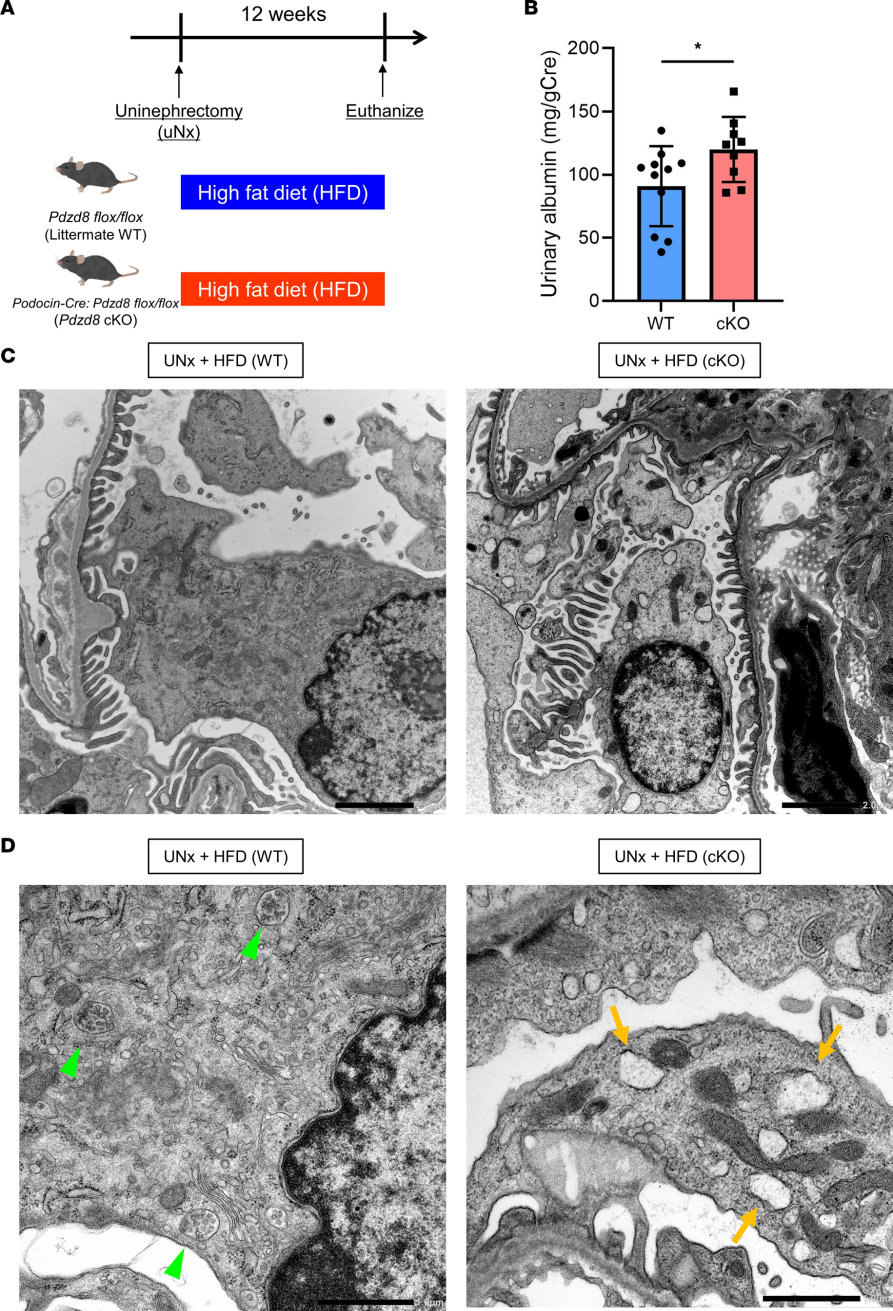
*Pdzd8* deletion leads to the emergence of fatty endosomes and exacerbates podocyte injury in an accelerated obesity–related kidney disease model. (**A**) The study design is shown (*n* = 9 or 11). (**B**) The urinary albumin levels are shown. (**C**) The electron microscope imaging with low magnification is shown. Scale bars: 2 μm. (**D**) The electron microscope imaging with high magnification is shown. Scale bars: 1 μm (green arrowheads: normal endosomes, orange arrows: abnormal fatty endosomes). Data are presented as mean ± SD. *P* value was determined by unpaired, 2-tailed Student’s *t* test. *P < 0.05.

To elucidate the impact of *Pdzd8* deletion–induced mitochondrial and endosomal abnormalities on podocyte injury, we established an accelerated obesity–related kidney disease model. WT and cKO mice were subjected to uninephrectomy (uNx) and exposed to HFD for 12 weeks (Figure 3A). Notably, significantly elevated urinary albumin levels, a marker of podocyte injury, were observed in uNx-HFD-cKO mice compared with that in uNx-HFD-WT mice (Figure 3B). Background data, including body weight, plasma cholesterol, plasma triglyceride, BUN, and plasma creatinine levels, did not significantly differ between uNx-HFD-WT and uNx-HFD-cKO groups (Supplemental Figure 3, A–E).

Optical microscopy imaging with PAS staining did not reveal a significant difference between uNx-HFD-WT and uNx-HFD-cKO mice (Supplemental Figure 3F). However, the number of Wilms tumor 1–positive (WT1-positive) cells per glomerulus was significantly reduced in uNx-HFD-cKO mice, compared with uNx-HFD-WT mice (Supplemental Figure 4). Moreover, electron microscopy imaging indicated numerous abnormal endosomes in the podocytes of uNx-HFD-cKO mice (Figure 3, C and D, orange arrows), labeled as “fatty endosomes,” suggesting a more advanced dysfunctional stage than those observed in HFD-cKO mice (Figure 1D). These endosomes were speculated to contain excessive lipids, prompting further investigation into their emergence mechanism.

### Pdzd8 knockdown inhibits mitochondrial and FAO activity in podocytes.

**Figure 4:**
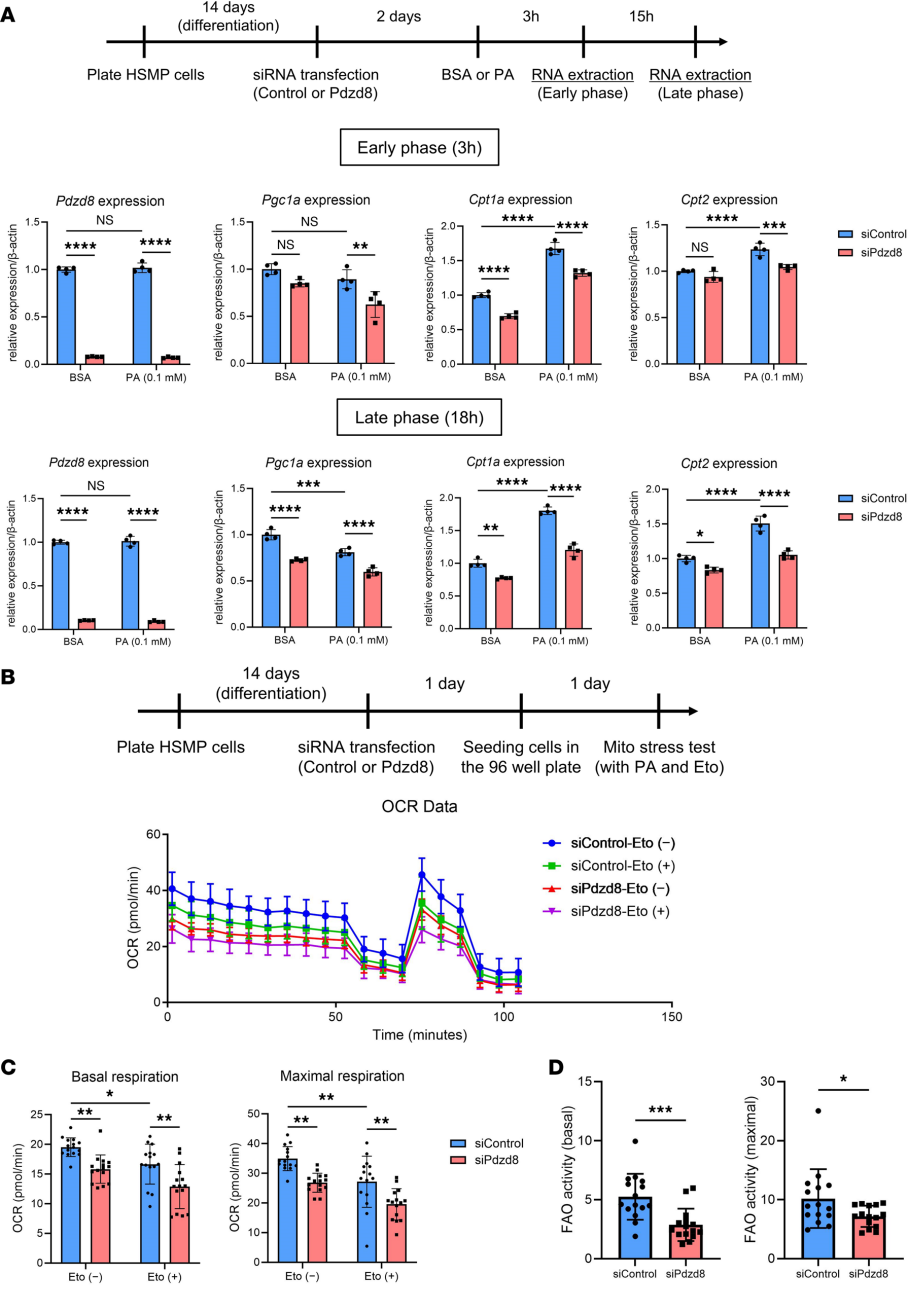
*Pdzd8* knockdown inhibits the activity of mitochondria and fatty acid oxidation (FAO) in podocytes. (**A**) The results of quantitative real-time PCR are shown (*n* = 4, each). HSMP, heat-sensitive mouse podocytes; BSA, bovine serum albumin; PA, palmitic acid; *Pgc1a*, peroxisome proliferator-activated receptor γ coactivator 1-α; *Cpt1a*, carnitine palmitoyltransferase-1a; *Cpt2*, carnitine palmitoyltransferase-2. (**B**–**D**) The results of Mito stress test are shown (*n* = 15, each). Eto, etomoxir; OCR, oxygen consumption rate. Data are presented as mean ± SD. *P* values were determined by 2-way ANOVA with Tukey’s multiple-comparison test (**A** and **C**) or unpaired, 2-tailed Student’s *t* test (**D**). *P < 0.05; **P < 0.01; ***P < 0.001; ****P < 0.0001. NS, not significant.

To corroborate the in vivo proteomic phenotype of mitochondrial abnormalities associated with PDZD8, we performed cell culture experiments using heat-sensitive mouse podocytes (HSMPs). Exposure of differentiated HSMPs to palmitic acid (PA) upregulated the mRNA expression of key FAO molecules, *Cpt1a* and *Cpt2*. *Pdzd8* knockdown using siRNA significantly inhibited the expression of these molecules at both early (3 hours) and late (18 hours) stages (Figure 4A). Proteomic analysis revealed a significant reduction in CPT1A protein levels after PA exposure (18 hours) following *Pdzd8* knockdown (Supplemental Figure 5A; jPOST repository accession number JPST002956). Additionally, *Pdzd8* knockdown decreased the expression of peroxisome proliferator-activated receptor γ coactivator 1-α (*Pgc1a*), a mitochondrial biosynthesis factor (Figure 4A). These phenotypes were consistent across multiple siRNA sequences, including *Pdzd8* siRNA (no. 2) (Supplemental Figure 5B).

We further performed a Mito Stress test with PA and etomoxir (CPT1 inhibitor) to assess mitochondrial oxygen consumption rate (OCR) and FAO activity (Figure 4B). *Pdzd8* knockdown significantly reduced basal and maximal OCR in both the absence and presence of etomoxir (Figure 4C). Moreover, *Pdzd8* knockdown inhibited FAO activity in both basal and maximal mitochondrial states (Figure 4D). Collectively, our findings suggest that *Pdzd8* knockdown inhibits mitochondrial activity, including that of FAO, in cultured podocyte cell lines.

We also performed flow cytometry analysis using Annexin V and propidium iodide (PI) to assess the rate of podocyte apoptosis. As a result, *Pdzd8* knockdown increased the apoptosis of PA-treated podocytes (Supplemental Figure 6). These data are consistent with the decrease in WT1-positive cells per glomerulus in uNx-HFD-cKO mice (Supplemental Figure 4).

### Pdzd8 knockdown results in a reduction in MERCSs in podocytes.

**Figure 5:**
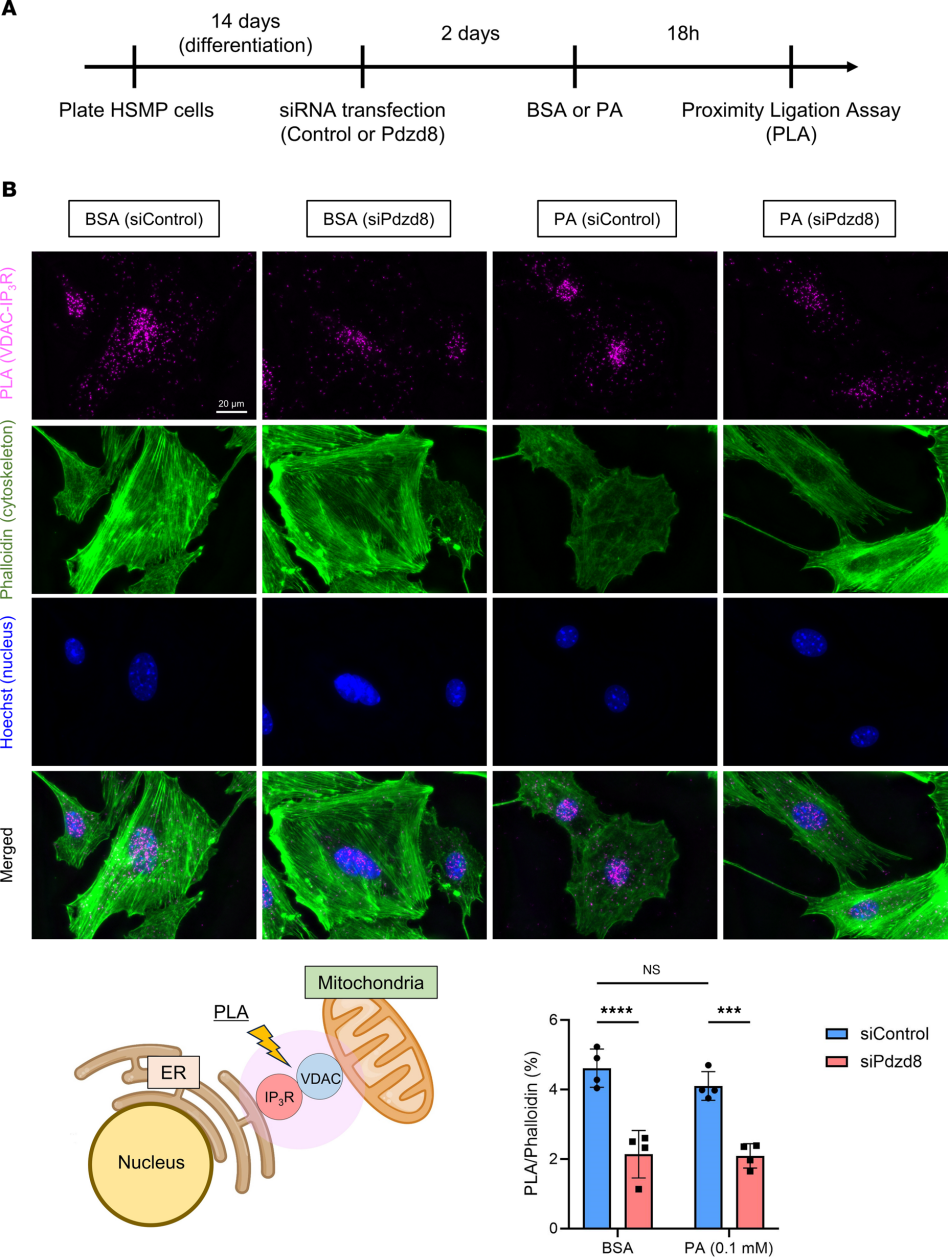
*Pdzd8* knockdown reduces mitochondria-ER contact sites (MERCSs) in podocytes. (**A**) The study design is shown. (**B**) Proximity ligation between VDAC and IP_3_R, phalloidin (cytoskeleton), Hoechst (nucleus), and merged pictures are shown. The process of calculating the ratio of proximity ligation signal to phalloidin is shown in Supplemental Figure 7 (*n* = 4, for each). Scale bar: 20 μm. VDAC, voltage-dependent anion channel; IP_3_R, inositol 1,4,5-trisphosphate receptor; PA, palmitic acid. Data are presented as mean ± SD. *P* values were determined by 2-way ANOVA with Tukey’s multiple-comparison test. ***P < 0.001, ****P < 0.0001. NS, not significant.

To explore interactions between the ER and mitochondria, we performed a proximity ligation assay (PLA), which helps assess the proximity between proteins at the MERCSs, utilizing voltage-dependent anion channel (VDAC) and inositol 1,4,5-trisphosphate receptor (IP_3_R) proteins representing the outer mitochondrial and ER membranes, respectively. The assay revealed a significant decrease in ER-mitochondria interactions in *Pdzd8*-knockdown podocytes compared with that in controls (Figure 5 and Supplemental Figure 7). This reduction in ER-mitochondria interactions induced by *Pdzd8* knockdown may contribute to mitochondrial inactivation.

### Lipids from cellular membranes accumulate in fatty endosomes in Pdzd8-knockdown podocytes following PA treatment.

**Figure 6:**
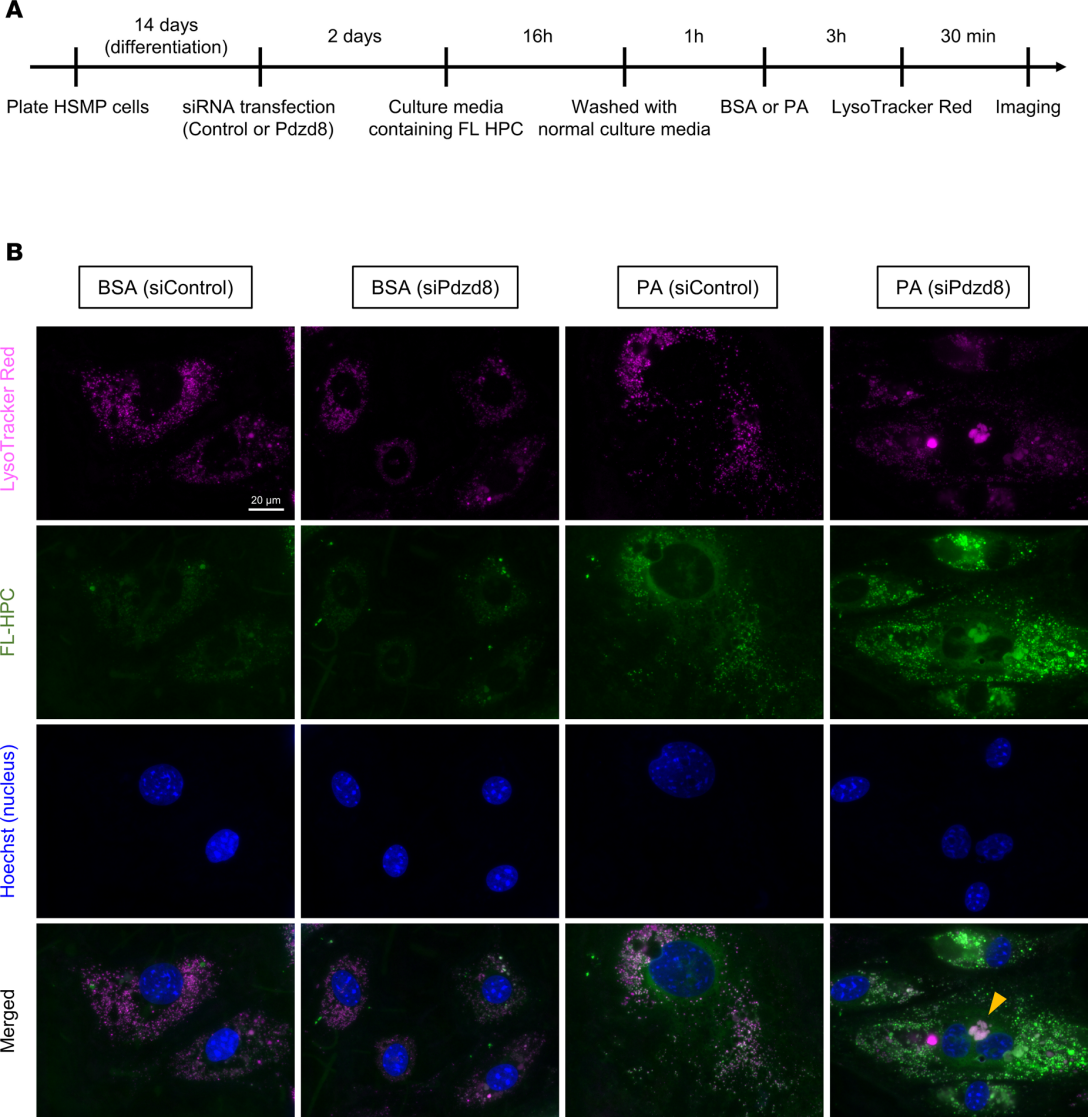
Lipids from cellular membranes accumulate in fatty endosomes in *Pdzd8*-knockdown podocytes following palmitic acid (PA) treatment. (**A**) The study design is shown. (**B**) LysoTracker Red, phosphatidylcholine fluorescence tagged at its fatty acid tail (FL HPC), Hoechst (nucleus), and merged pictures are shown. Scale bar: 20 μm (orange arrowhead: fatty endosomes).

To explore the origin of fatty endosomes, we performed an FA pulse-chase assay using FA tail–tagged phosphatidylcholine fluorescence (FL HPC) (Figure 6A) (12). Initial labeling of cultured podocytes with FL HPC, which integrates into various cellular membranes, revealed significantly higher FL HPC and LysoTracker Red colocalization in PA-treated podocytes compared with that in BSA-treated podocytes (Figure 6B), suggesting that PA treatment mobilized lipids from cellular membranes to endosomes. Notably, enlarged endosomes colocalized with FL HPC were observed in PA-treated *Pdzd8*-knockdown podocytes (Figure 6B, orange arrowhead), resembling fatty endosomes observed in the podocytes of uNx-HFD-cKO mice (Figure 3, C and D). Moreover, at a later stage (PA treatment for 18 hours), RAB7-labeled endosomes significantly accumulated in *Pdzd8*-knockdown podocytes compared with that in the negative control siRNA–treated group (Supplemental Figures 8 and 9). Thus, lipids from cellular membranes may accumulate in fatty endosomes in *Pdzd8*-knockdown podocytes after PA treatment.

### Pdzd8 knockdown induces glucosylceramide accumulation in podocytes.

**Figure 7:**
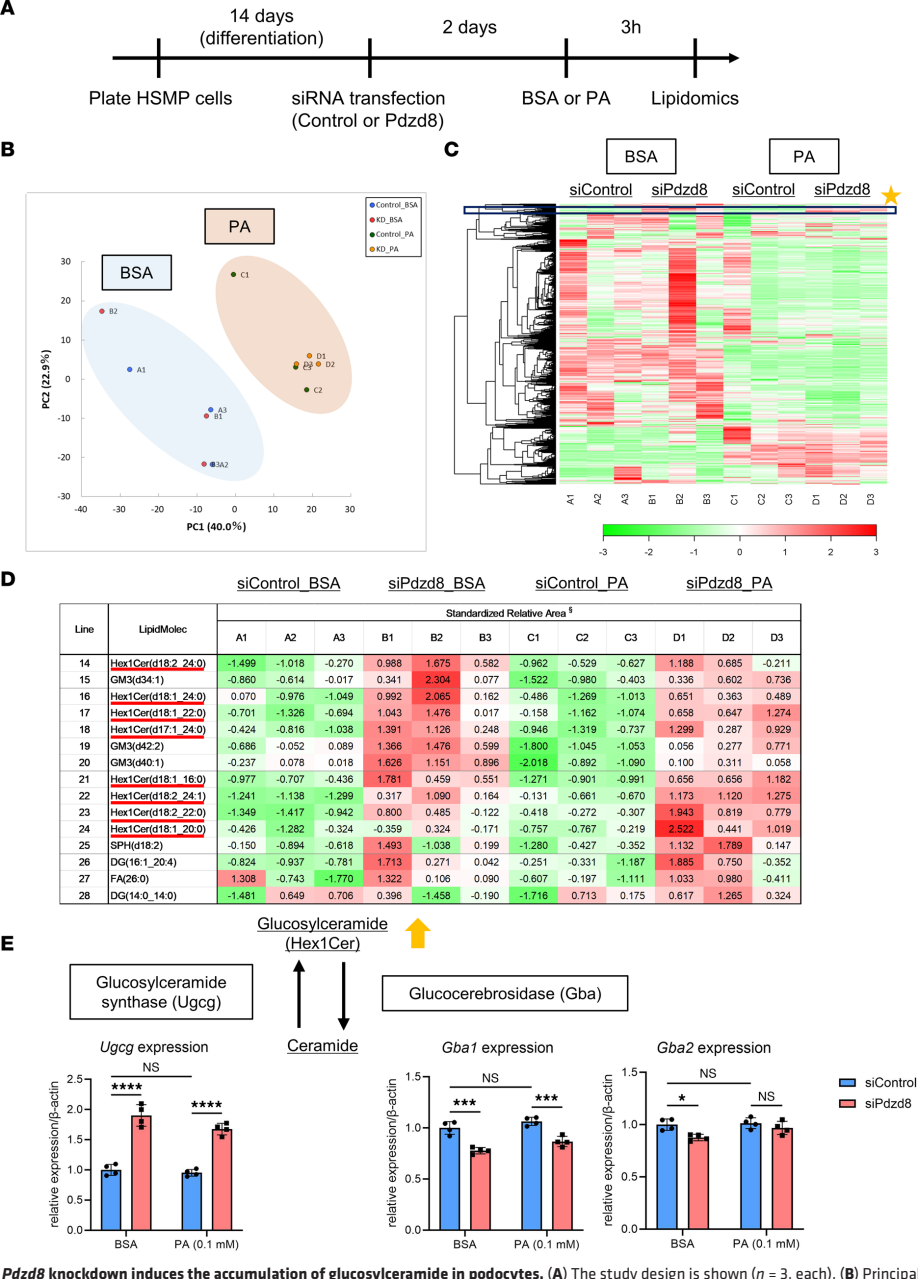
*Pdzd8* knockdown induces the accumulation of glucosylceramide in podocytes. (**A**) The study design is shown (*n* = 3, each). (**B**) Principal component analysis (PCA) plot of the nontargeted lipidomics of cultured podocytes is shown. (**C**) Heatmap of the identified lipids is shown. (**D**) The excerpt of the lipids that are increased by *Pdzd8* knockdown (the orange star in **C**). (**E**) The expression of enzymes for the production and degradation of glucosylceramide is shown (*n* = 4, each). Hex1cer, glucosylceramide; GM3, gangliosides; SPH, sphingosine; DG, diglyceride; FA, fatty acid; *Ugcg*, UDP-glucose ceramide glucosyltransferase; *Gba1*, glucosylceramidase β1; *Gba2*, glucosylceramidase β2. Data are presented as mean ± SD. *P* values were determined by 2-way ANOVA with Tukey’s multiple-comparison test. *P < 0.05, ***P < 0.001, ****P < 0.0001. NS, not significant.

To identify the lipid types accumulating in fatty endosomes, we performed nontargeted lipidome analysis on cultured podocytes (Figure 7A and Supplemental Table 3). While the lipid profiles differed between the BSA and PA treatment groups, no significant differences were observed in lipid profiles between control and *Pdzd8*-knockdown podocytes (Figure 7B). However, examination of upregulated lipids attributable to *Pdzd8* knockdown (Figure 7C) revealed enrichment in glucosylceramide (Hex1Cer) (Figure 7D).

As glucosylceramide production involves regulation by glucosylceramide synthase (UDP-glucose ceramide glucosyltransferase, UGCG), and glucosylceramide degradation involves regulation by glucosylceramidase β1 (GBA1) and glucosylceramidase β2 (GBA2), their expression was investigated. Quantitative real-time PCR showed that *Pdzd8* knockdown increased *Ugcg* (synthesis) expression and decreased *Gba1* (degradation in endosomes) expression (Figure 7E). In contrast, *Gba2* (degradation at the ER) expression did not differ between control and *Pdzd8*-knockdown podocytes (Figure 7E). Consistently, in vivo, *Pdzd8* knockout tended to increase UGCG expression and decreased GBA2 expression in the isolated glomeruli of an HFD model (Supplemental Figure 10). Thus, *Pdzd8* knockdown induces glucosylceramide accumulation in podocytes by activating their synthesis and reducing their degradation in endosomes, which might at least partly contribute to the emergence of fatty endosomes in *Pdzd8*-deleted podocytes.

We also performed *Gba1* knockdown experiments to examine the effects of glucosylceramide accumulation on podocyte conditions. Flow cytometry analysis using Annexin V and PI showed that *Gba1* knockdown increased the apoptosis of podocytes after 18-hour PA exposure (Supplemental Figure 11). Moreover, *Gba1* knockdown reduced the expression of *Pgc1a*, *Cpt1a*, and *Cpt2* in podocytes (Supplemental Figure 11), suggesting that glucosylceramide accumulation inhibited mitochondrial and FAO activity.

## Discussion

**Figure 8:**
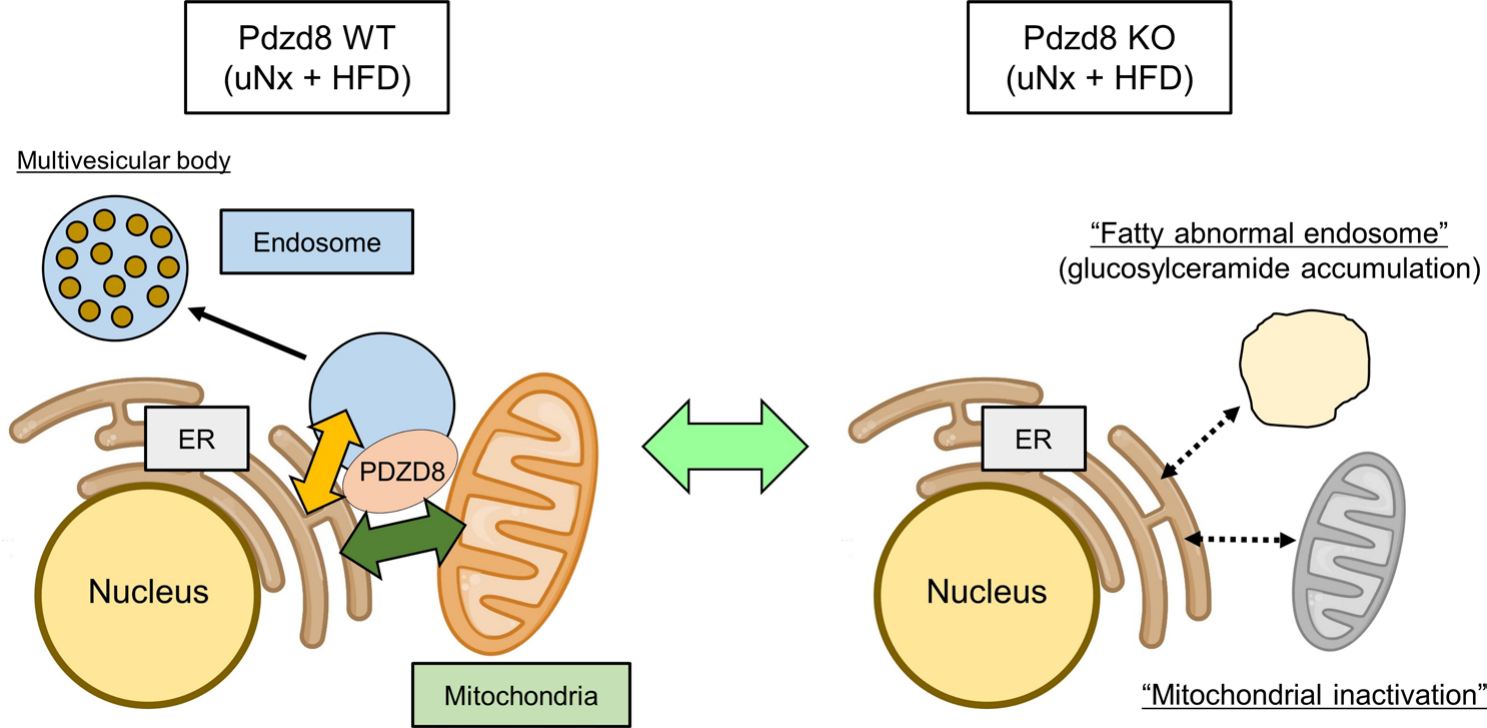
Scheme showing the protective role of organelle communications among the ER, mitochondria, and endosomes in podocyte lipotoxicity. Our findings demonstrate that PDZD8 serves as a critical organelle-tethering factor, maintaining mitochondrial and endosomal homeostasis during podocyte lipotoxicity.

In this study, we demonstrated that the organelle-tethering factor, PDZD8, plays a crucial role in maintaining mitochondrial and endosomal homeostasis during podocyte lipotoxicity (Figure 8). *Pdzd8* deficiency inhibits mitochondrial activity, including FAO, leading to the accumulation of fatty endosomes. This accumulation is partly attributed to the acceleration of glucosylceramide production and the deceleration of its degradation during obesity-related kidney disease. These findings highlight the significant contribution of 3-way organelle communication among the ER, mitochondria, and endosomes to podocyte homeostasis.

During the progression of diabetic kidney disease (DKD), organelle stressors, such as excessive ATP production (mitochondrial stress) and maladaptive unfolded protein response (UPR; ER stress), occur simultaneously in kidney tissues (13, 14). Furthermore, the reduced intraflagellar transport protein 88 (IFT88) expression in cisplatin-induced acute kidney injury (AKI) shortens primary cilia and leads to mitochondrial dysfunction (15). These findings emphasize the importance of detailed analysis of organelle communication in understanding organelle stress in kidney disease.

Extensive research on MERCSs in various diseases suggests that their disruption contributes to insulin resistance in the liver (16) and muscles (17). Overexpression of mitofusin-2 (18) and glucose-regulated protein 75 (19), which are pivotal components of MERCS, in hepatocytes effectively prevents PA-induced insulin signaling deficiency. The role of MERCSs extends to kidney disease progression, where their disruption occurs at the early stages of gentamicin-induced AKI. This disruption precedes downstream activation of the UPR and subsequent cell death (20). Furthermore, increased expression of the ER-resident protein reticulon-1A contributes to tubular injury, tubulointerstitial fibrosis, and a decline in kidney function during the early stages of DKD by regulating ER-mitochondria contacts (21). Additionally, urine metabolome analysis has identified lysophosphatidylcholine (LPC) accumulation as a negative prognostic factor for DKD. The accumulation of LPC in proximal tubular cells decreases MERCSs and leads to cell death (22).

Our study introduces what we believe is a novel perspective by emphasizing the significance of the 3-way organelle-tethering factor and elucidates that the communication among the ER, mitochondria, and endosomes significantly contributes to maintaining podocyte homeostasis. However, the impact of *Pdzd8* deletion on albuminuria was less pronounced than anticipated despite the evident organelle dysfunctions observed, including the presence of fatty endosomes in *Pdzd8*-deleted podocytes.

There may be 3 reasons for this observed discrepancy. First, glomerular filtration is a passive process and does not require a substantial amount of ATP, rendering podocytes less sensitive to mitochondrial dysfunction (23). Second, in terms of protein degradation, the ubiquitin-proteasome system might play a more pivotal role than the autophagy-endosome system in maintaining intracellular homeostasis in podocytes (24). While the specific deficiency of autophagy in podocytes produced no discernible histological phenotype in young mice (25, 26), the impairment of the podocyte-specific proteasome led to apoptosis and the development of glomerulosclerosis at a young age (24). Hence, the ubiquitin-proteasome system might compensate for the endosomal deficiency induced by *Pdzd8* deletion. Third, *Pdzd8* deficiency results in the accumulation of glucosylceramide. Cardiolipin accumulation is reportedly implicated in podocyte lipotoxicity (27). However, our lipidome analysis indicated that *Pdzd8* deletion did not induce cardiolipin accumulation, but rather led to the accumulation of glucosylceramide in podocytes, which is similar to that in Gaucher disease. Gaucher disease is a rare, inherited metabolic disorder in which deficiency of GBA1 results in glucosylceramide accumulation throughout the body, particularly in the bone marrow, spleen, and liver (28). In contrast to Fabry disease, patients with Gaucher disease typically do not experience rapid progression of kidney disease, suggesting that glucosylceramide accumulation might not significantly impact podocyte homeostasis.

The detailed mechanism underlying the fact that *Pdzd8* deletion increases the expression of *Ugcg* and decreases the expression of *Gba1* in podocytes remains elusive. However, a previous study showed that UGCG directly interacted with reticulon-1 in MERCSs (29). The increased expression of *Ugcg* in *Pdzd8*-knockdown podocytes might be attributable to the disruption of MERCSs. As GBA1 is expressed in endosomes, the decreased expression of *Gba1* in *Pdzd8*-knockdown podocytes might be induced by endosomal dysfunction.

This study has the following limitations: First, our study focused on PDZD8 functions rather than organelle tethering. We acknowledge that PDZD8 may have additional roles beyond being a 3-way organelle-tethering factor among the ER, mitochondria, and endosomes. Thus, we cannot conclude whether the phenotypes of podocyte-specific *Pdzd8* deletion are primarily attributable to the disruption of organelle communications. However, the knockdown of inositol 1,4,5-trisphosphate receptor type 3 (*Itpr3*), another MERCS factor, also induced mitochondrial inactivation in podocytes (Supplemental Figure 12). Thus, the role of PDZD8 in mitochondrial function might be associated with the disruption of MERCSs. Second, we performed whole-cell lipidomics to identify the origin of fatty endosomes. Although isolating these endosomes and performing lipidomic analysis is imperative to comprehensively investigate their role, maintaining consistent purity in the organelles isolated from podocytes with different injury statuses is technically complex. Thus, we cannot conclude whether glucosylceramide was specifically increased within the fatty endosomes or in the other cellular fractions.

Nevertheless, our findings demonstrate that PDZD8 serves as a critical organelle-tethering factor, maintaining mitochondrial and endosomal homeostasis during podocyte lipotoxicity (Figure 8). Its deficiency inhibits mitochondrial activity, including FAO, induces fatty endosome accumulation by accelerating the production of glucosylceramide and decelerating its degradation, and contributes to obesity-related kidney disease. Further research on additional organelle-tethering factors is required to comprehensively elucidate the role of organelle communication in podocyte homeostasis.

## Methods

### Sex as a biological variable.

Our study examined male mice because male animals exhibited less variability in phenotype.

### Animal experiments.

Male podocyte-specific *Pdzd8*-cKO mice (background: C57BL/6, 9–10 weeks of age, 20–25 g) were generated by crossbreeding podocin-Cre (30) and *Pdzd8^fl/fl^* mice (31). Genotyping was performed by tail PCR using published primers (Supplemental Figure 1, A and B, and Supplemental Table 4). HFD-60 (Oriental Yeast Co., Ltd.) was used as the HFD (calorie ratio of FAs is 62.2%). CE-2 (Clea Japan, Inc.) was used as the ND (calorie ratio of FAs is 4.77%).

### Uninephrectomy.

Mice were anesthetized by the intraperitoneal administration of 0.3 mg/kg medetomidine, 5 mg/kg butorphanol, and 4 mg/kg midazolam. Left-side unilateral nephrectomy was performed, and the wound was sutured.

### Kidney function and histological analyses.

Plasma of heparinized blood was separated by centrifugation at 7,000*g* for 5 minutes at room temperature. Plasma cholesterol, plasma triglyceride, BUN, and plasma creatinine levels were measured by SRL Inc. Urinary albumin and creatinine levels were measured by Oriental Yeast Co., Ltd. For histological analyses, the kidney tissues were fixed in Mildform 10N (133-10311, Wako), dehydrated, and embedded in paraffin. The paraffin-embedded samples were cut into 3-μm-thick sections and stained with PAS. As for the WT1 staining, antigen retrieval was carried out using microwave irradiation (500 W, 5 minutes, twice). Endogenous peroxidase was inactivated by treatment with 3% hydrogen peroxide. The kidney slices were incubated with an anti-WT1 antibody (1:300; ab89901, Abcam) at 4°C overnight. After incubated with an HRP-conjugated secondary antibody (Histo-fine simple stain mouse MAX-PO, 414341, Nichirei), diaminobenzidine (DAB) reactions were performed using ImmPACT DAB peroxidase substrate (SK-4105, Vector Laboratories) according to the manufacturer’s instructions.

### Electron microscopy.

The kidney tissues were fixed with 2% paraformaldehyde and 2.5% glutaraldehyde in 0.1 M phosphate buffer (pH 7.4). The samples were postfixed with 1% osmium tetroxide, dehydrated, and embedded in Epok 812. Ultrathin sections were stained with uranyl acetate and lead citrate. The samples were observed using a JEM-1400 Flush electron microscope (JEOL Ltd.).

### Glomerular isolation.

Mouse glomeruli were isolated using a previously described method, with some modifications (30). Briefly, we perfused magnetic Dynabeads M-450 (DB14013, Invitrogen) through the left ventricle of the heart. After removal, kidneys were minced into small pieces, digested by collagenase A (2 mg/mL; 10103586001, Roche) and DNase I (200 units/mL; 04716728001, Roche), and filtered through a 100 μm cell strainer with sterile PBS. After washing several times, the glomeruli were collected using a magnet. The purity of glomeruli was confirmed to be 95% in each sample by phase-contrast microscopy.

### Proteomics.

Glomerular and cellular proteomics was performed and analyzed by the Kazusa DNA Research Institute (32–34). The PCA and the comparison between groups were performed using Perseus (https://maxquant.net/perseus/) (35). PCA decomposes the original data into a set of new variables that are linear combinations of the original variables. Component 1 is the line that best accounts for the shape of the point swarm. It represents the maximum variance direction in the data. Component 2 is oriented such that it reflects the second largest source of variation in the data while being orthogonal to component 1. The definition of differentially expressed proteins was |log_2_(fold change)| greater than 1 and a *P* value of less than 0.05. The GO analysis of differentially expressed proteins was performed using Metascape (https://metascape.org/) (36).

### Lipidomics.

Cellular lipidomics was conducted according to the Non-targeted Lipidome Scan package (Lipidome Lab), using liquid chromatography–orbitrap mass spectrometry based on methods described previously (37, 38). Hierarchical cluster analysis and PCA were performed by Human Metabolome Technologies, Inc. (HMT), using HMT’s proprietary MATLAB and R programs, respectively (39).

### RNA isolation and quantitative real-time PCR.

Total mRNA was isolated from cultured cells or mouse tissue samples using the RNeasy Mini Kit (74106, Qiagen), and 50 ng mRNA in a total reaction volume of 20 μL was reverse transcribed using PrimeScript RT Master Mix (Takara Bio) according to the manufacturers’ protocols.

The complementary DNA (cDNA) generated was used as the template for quantitative real-time PCR that was performed with Fast SYBR Green Master Mix (Thermo Fisher Scientific) on a StepOnePlus real-time PCR system (Thermo Fisher Scientific). *Actb* (β-actin) was used as the reference gene. Relative gene expression levels were calculated from cycle threshold (Ct) values (2^–ΔΔCt^) (40). The primer sequences used in this study are listed in Table 1.

### Cell culture.

Conditionally immortalized HSMPs were generated and characterized as described previously (30, 41, 42). The cells were grown in RPMI 1640 media with HEPES (189-02145, Wako) containing 10% FBS (Thermo Fisher Scientific) and 1 mmol/L sodium pyruvate (Wako). To passage cells, podocytes were grown under permissive conditions (33°C in the presence of 50 U/mL IFN-γ). For podocytes to acquire differentiation and quiescence resembling the in vivo phenotype, cells were grown under restrictive conditions at 37°C in 95% air/5% CO_2_ without IFN-γ for 14 days. All experiments were performed using podocytes under growth-restricted, differentiated conditions.

### RNA interference.

Gene expression was suppressed using Silencer Select Pre-designed siRNAs (Thermo Fisher Scientific) against mouse *Pdzd8* (s98833 as no. 1 and s98834 as no. 2), mouse *Gba1* (s66492), and mouse *Itpr3* (s68521). A supplier-matched Silencer Select Negative Control No. 1 siRNA (4390843, Thermo Fisher Scientific) was used as the negative control. The siRNAs (5 nM) were introduced into mouse podocytess using Opti-MEM I Reduced Serum Medium (31985070, Thermo Fisher Scientific) and Lipofectamine RNAiMAX transfection reagent (13778150, Thermo Fisher Scientific) according to the manufacturer’s instructions.

### Analysis of mitochondrial function and FAO.

The intracellular bioenergetic profiles of cells were determined with a Seahorse XFe96 extracellular flux analyzer (Agilent Technologies), using the Mito Stress Test Kit (103015–100, Agilent Technologies) according to the manufacturer’s protocol (15). The concentrations of glucose, pyruvate, and glutamine in the assay media were 10 mM, 1 mM, and 2 mM, respectively. The concentrations of oligomycin, carbonylcyanide-4-trifluoromethoxyphenylhydrazone (FCCP), and antimycin A/rotenone during Mito Stress tests were 2 μM, 0.5 μM, and 1 μM, respectively. To measure FAO activity, PA-conjugated BSA (102720–100, Agilent Technologies) and etomoxir (40 μM), an inhibitor of CPT1, were added to the medium before the assay. FAO activity was defined as the etomoxir-induced decrease in basal and maximal OCR.

### Flow cytometry analysis.

Apoptosis assay was performed using an Annexin V-FITC Apoptosis Detection Kit (15342-54, Nacalai). The flow cytometry analysis was conducted using the CytoFLEX System (Beckman Coulter).

### Immunofluorescent staining.

For immunofluorescent staining, the cells were seeded at 8 × 10^4^ cells/well in 35-mm glass-base dishes coated with collagen (D11134H, Iwaki). After aspiration of the culture medium, the cells were washed with PBS and then fixed with 4% paraformaldehyde solution (163–20145, Wako) for 10 minutes. The fixed cells were washed 3 times with PBS and were then permeabilized with 0.3% Triton X-100–containing PBS for 10 minutes. The cells were blocked with 1% BSA in PBS for 30 minutes. After incubation with rabbit monoclonal anti-RAB7 [EPR7589] (1:500; ab137029, Abcam) for 1 hour, the cells were washed 3 times with PBS and then incubated with goat anti–rabbit IgG (H+L) cross-adsorbed secondary antibody, Texas Red (1:1000; T-2767, Thermo Fisher Scientific) for an additional hour. Thereafter, the cells were once again washed 3 times with PBS. Cytoskeletons were stained with Alexa Fluor 488–phalloidin (A12379, Thermo Fisher Scientific) and nuclei were stained with Hoechst 33342 (B2261, Sigma-Aldrich). All procedures were performed at room temperature. The samples were observed using a BZ-X700 fluorescence microscope (KEYENCE). Image analysis was performed using ImageJ software (43).

### PLA.

For PLA, the Duolink In Situ Red Starter Kit Mouse/Rabbit (DUO92101, Sigma-Aldrich) was used (44, 45). The cells were seeded at 8 × 10^4^ cells/well in 35-mm glass-base dish coated with collagen (D11134H, Iwaki). After aspiration of the culture medium, the cells were washed with PBS and then fixed with 4% paraformaldehyde solution (163–20145, Wako) for 10 minutes. The fixed cells were washed 3 times with PBS and were then permeabilized with 0.3% Triton X-100–containing PBS for 30 minutes. Blocking was performed using Duolink blocking solution for 1 hour at 37°C. Incubations with primary antibodies rabbit polyclonal anti-VDAC1 (1:100; 55259-1-AP, Proteintech) and mouse monoclonal anti–IP_3_R-1 (E-8) (1:100; sc-271197, Santa Cruz Biotechnology) (both diluted in Duolink Antibody diluent) were performed overnight at 4°C in a humidity chamber. Then, the cells were rinsed with Wash Buffer A. Next, PLUS and MINUS secondary PLA probes, both rabbit and mouse immunoglobulins diluted in Duolink Antibody diluent, were added for 1 hour at 37°C. The incubation was followed by 5-minute washes with 2 changes of Wash Buffer A. After this, the dishes were incubated with the Duolink ligation mix for 30 minutes at 37°C and thereafter washed with 2 changes of Wash Buffer A for 5 minutes each. The Duolink amplification mix was then applied to the dishes for 100 minutes at 37°C. Subsequently, the dishes were washed twice for 10 minutes each time with Wash Buffer B. Cytoskeletons were stained with Alexa Fluor 488–phalloidin (A12379, Thermo Fisher Scientific) and nuclei were stained with Hoechst 33342 (B2261, Sigma-Aldrich). The samples were observed using a BZ-X700 fluorescence microscope (KEYENCE). Image analysis was performed using ImageJ software (43).

### Fluorescent FA pulse-chase assay.

Fluorescent FA pulse-chase assay was performed as previously described, with slight modification (12). Mouse podocytes were incubated with the culture media containing 2 mM β-BODIPY FL C_12_-HPC (FL HPC) (Invitrogen) for 16 hours. Cells were then washed 3 times with the normal culture media, incubated for 1 hour to allow the fluorescent lipids to incorporate into cellular membranes, and then chased for the indicated times under BSA or PA-BSA (0.1 mM) treatment. FL-HPC–loaded and chased cells were stained with 50 nM LysoTracker Red DND-99 for 30 minutes at 37°C and their nuclei were stained with Hoechst 33342 (B2261, Sigma-Aldrich). The samples were observed using a BZ-X700 fluorescence microscope (KEYENCE).

### Statistics.

The data are expressed as mean ± SD. Student’s *t* test (unpaired, 2-tailed) was used for the comparisons between 2 subgroups. For multiplex comparisons, a 2-way analysis of variance (ANOVA) with a post hoc Tukey’s multiple-comparison test was applied. A *P* value of less than 0.05 was considered statistically significant. All statistical analyses were performed using Prism 10 software (GraphPad Software, Inc.).

### Study approval.

All animal experiments were approved by the University of Tokyo Institutional Review Board (approval numbers P20-098 and H22-184). All animal procedures were performed according to the NIH *Guide for the Care and Use of the Laboratory Animals* (National Academies Press, 2011).

### Data availability.

The glomerular and cellular proteomics data supporting the findings of this study are openly available in the jPOST repository (https://repository.jpostdb.org/) (46) under accession numbers JPST002955 and JPST002956, respectively. Values for all data points in graphs are reported in the supplemental Supporting Data Values file.

## Author contributions

SH, MN, and RI designed the study. SH performed most of the experiments. YH provided technical advice. YT, CK, QL, MS, YAH, and JS provided technical support for the experiments. NK and TK provided conceptual advice. SH wrote the original manuscript. All authors revised the manuscript and approved the final version.

## Supplementary Material

Supplemental data

Supplemental table 1

Supplemental table 2

Supplemental table 3

Supplemental table 4

Supporting data values

## Figures and Tables

**Table 1:**
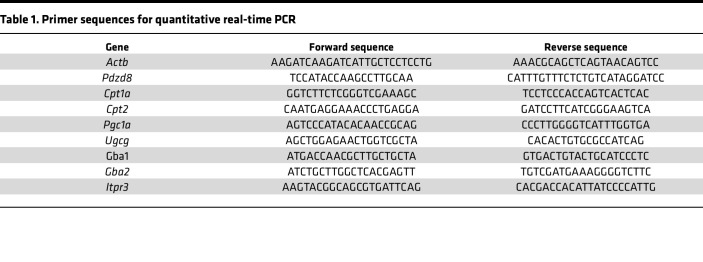
Primer sequences for quantitative real-time PCR

## Version 1 08/08/2024

In-Press Preview

## Version 2 09/24/2024

Electronic publication
