# Supplementary material for: Organelle communication maintains mitochondrial and endosomal homeostasis during podocyte lipotoxicity
Source: JCI Insight. 2024 Aug 8;9(18):e182534. doi: 10.1172/jci.insight.182534 (PMC11457848; doi:10.1172/jci.insight.182534)
Supplement: Supplemental data [file jciinsight-9-182534-s039.pdf]

Supplementary Materials

(Organelle communication maintains mitochondrial and endosomal homeostasis during podocyte lipotoxicity)

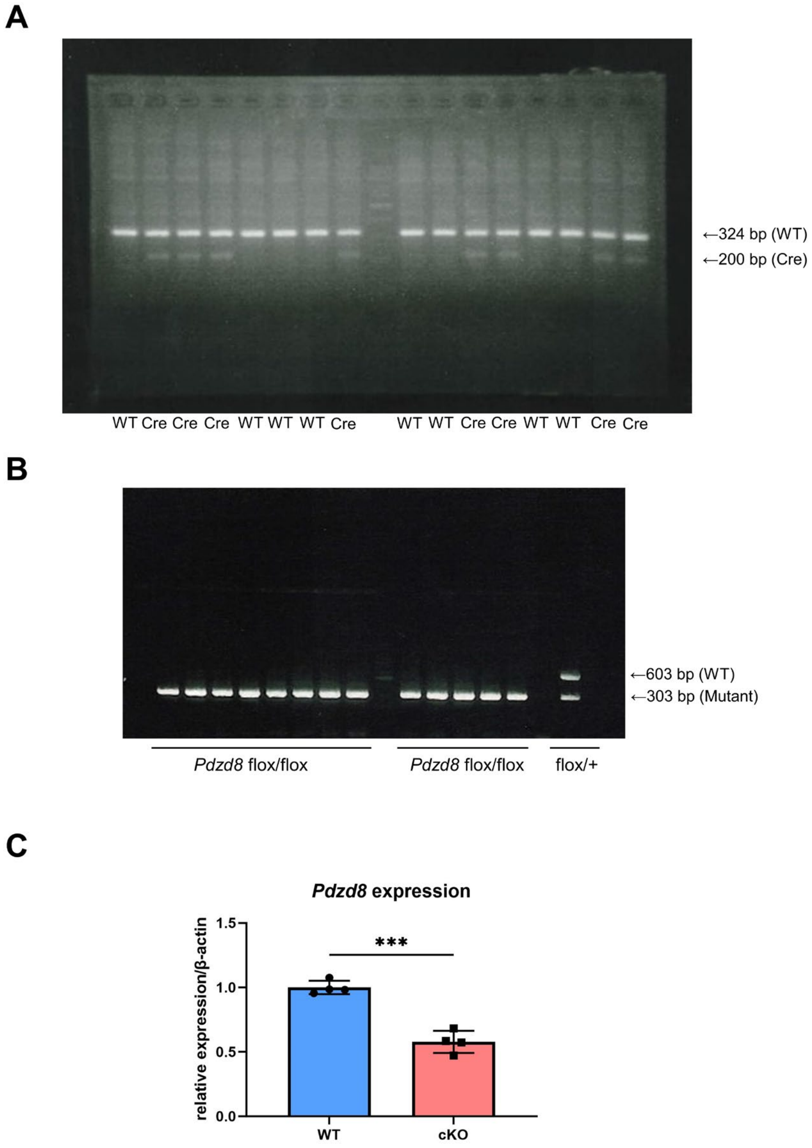

Supplementary Figure S1. Generation of podocyte-specific *Pdzd8* knockout mice

(A) Genotyping of Podocin-Cre is shown. (B) Genotyping of *Pdzd8* flox is shown. (C) The mRNA expressions of *Pdzd8* in the isolated glomeruli are significantly reduced in Podocin-Cre: *Pdzd8* flox/flox mice (cKO) compared with *Pdzd8* flox/flox mice (WT). As *Pdzd8* is expressed in various cell types including vascular endothelial cells as well as podocytes, the deletion rate in glomeruli is not 100%. Data are presented as mean ± SD. *P*-value is determined by unpaired Student's *t* test. ***$P < 0.001$.

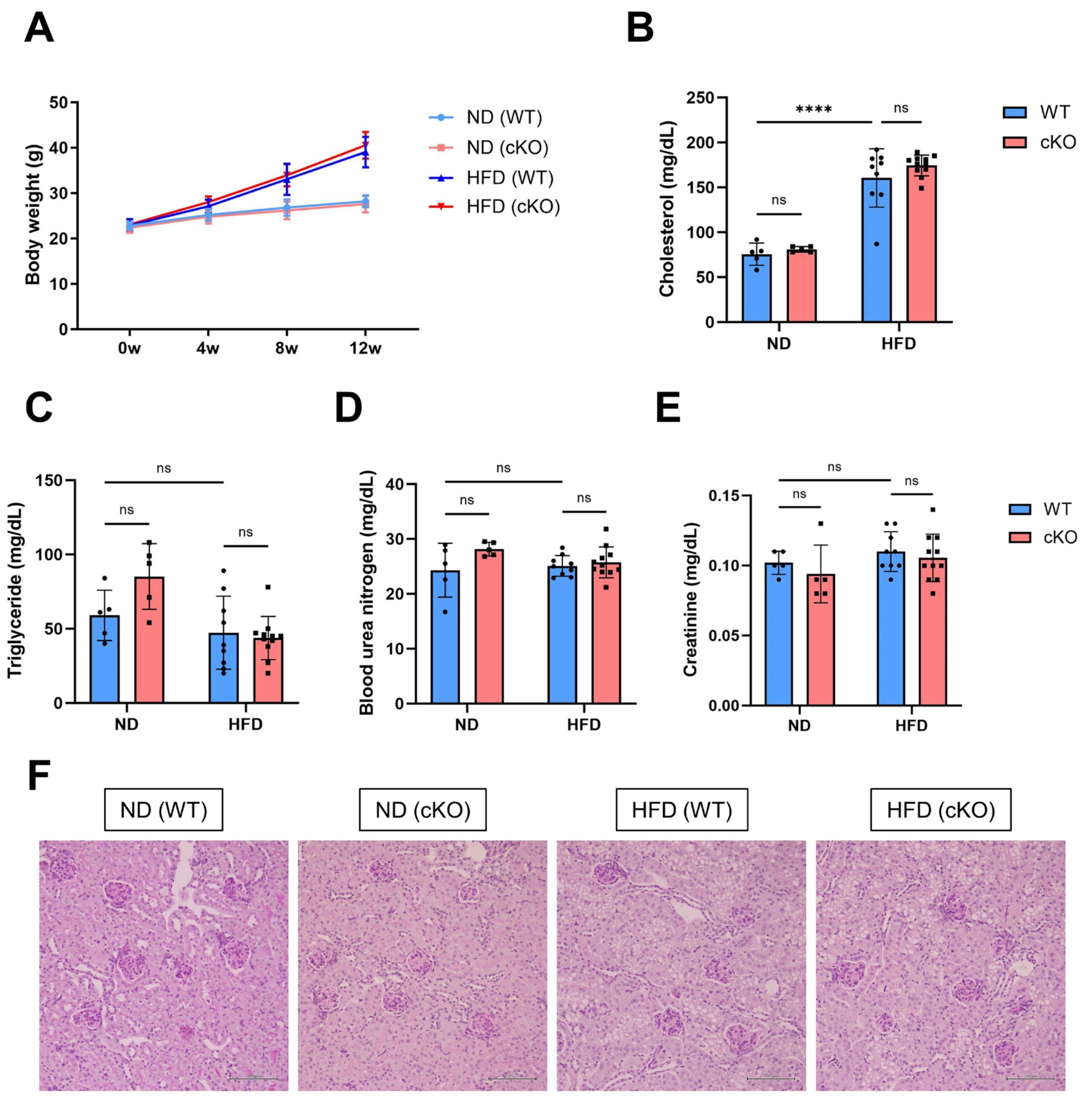

Supplementary Figure S2. Background data of an obesity related kidney disease model using podocyte-specific *Pdzd8* knockout mice

(A) Body weights, (B) plasma cholesterol levels, (C) plasma triglyceride levels, (D) blood urea nitrogen (BUN) levels, and (E) plasma creatinine levels are shown (ND: n = 5, HFD: n = 9 or 11). (F) The optical microscope imaging with PAS staining is shown. Scale bar = 500 μm. Data are presented as mean ± SD. *P*-values are determined by 2-way ANOVA with Tukey's multiple-comparison test. *****P* < 0.0001, ns, not significant.

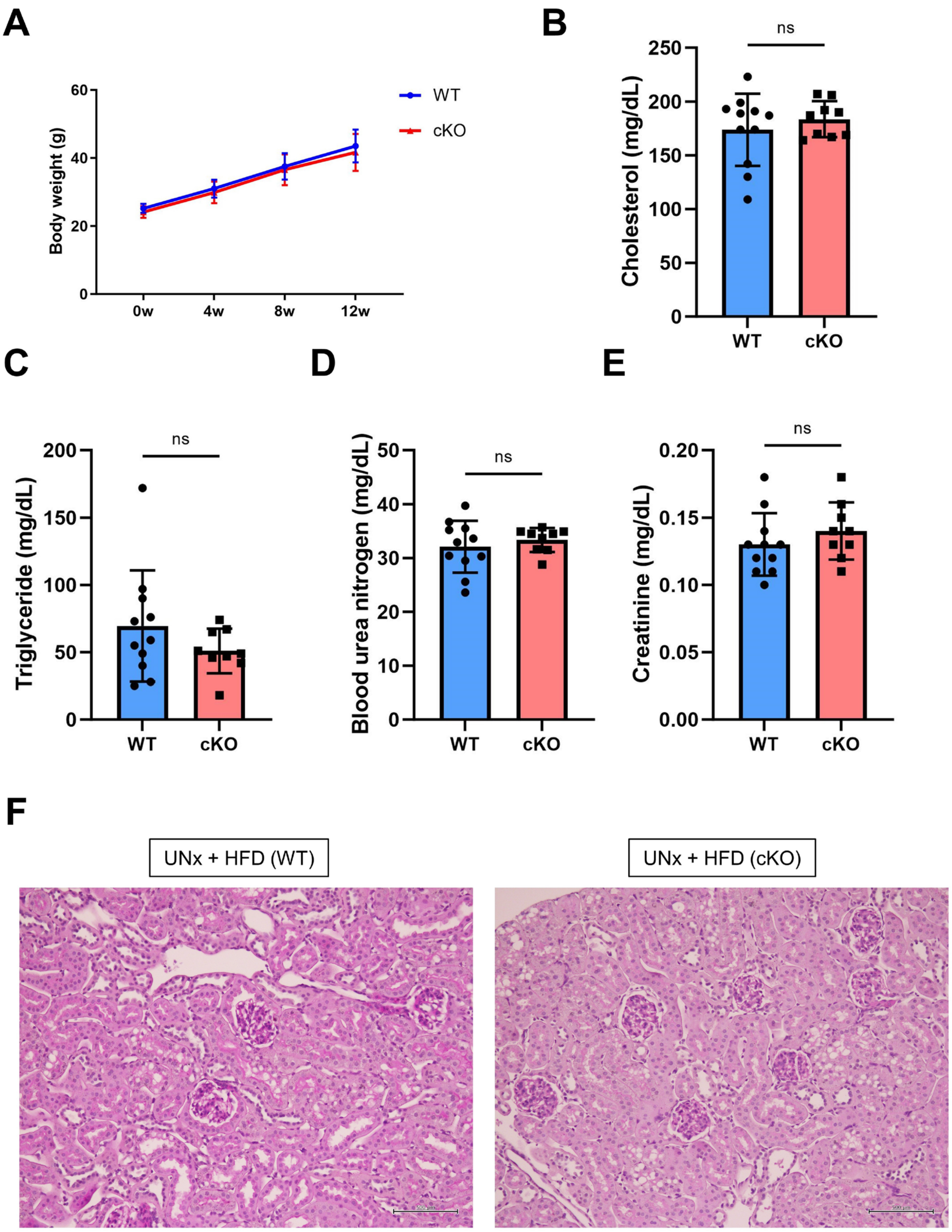

Supplementary Figure S3. Background data of an accelerated obesity related kidney disease model using podocyte-specific *Pdzd8* knockout mice

(A) Body weights, (B) plasma cholesterol levels, (C) plasma triglyceride levels, (D) blood urea nitrogen (BUN) levels, and (E) plasma creatinine levels are shown (n = 9 or 11). (F) The optical microscope imaging with PAS staining is shown. Scale bar = 500 μm. Data are presented as mean ± SD. *P*-values are determined by unpaired Student’s *t* test. ns, not significant.

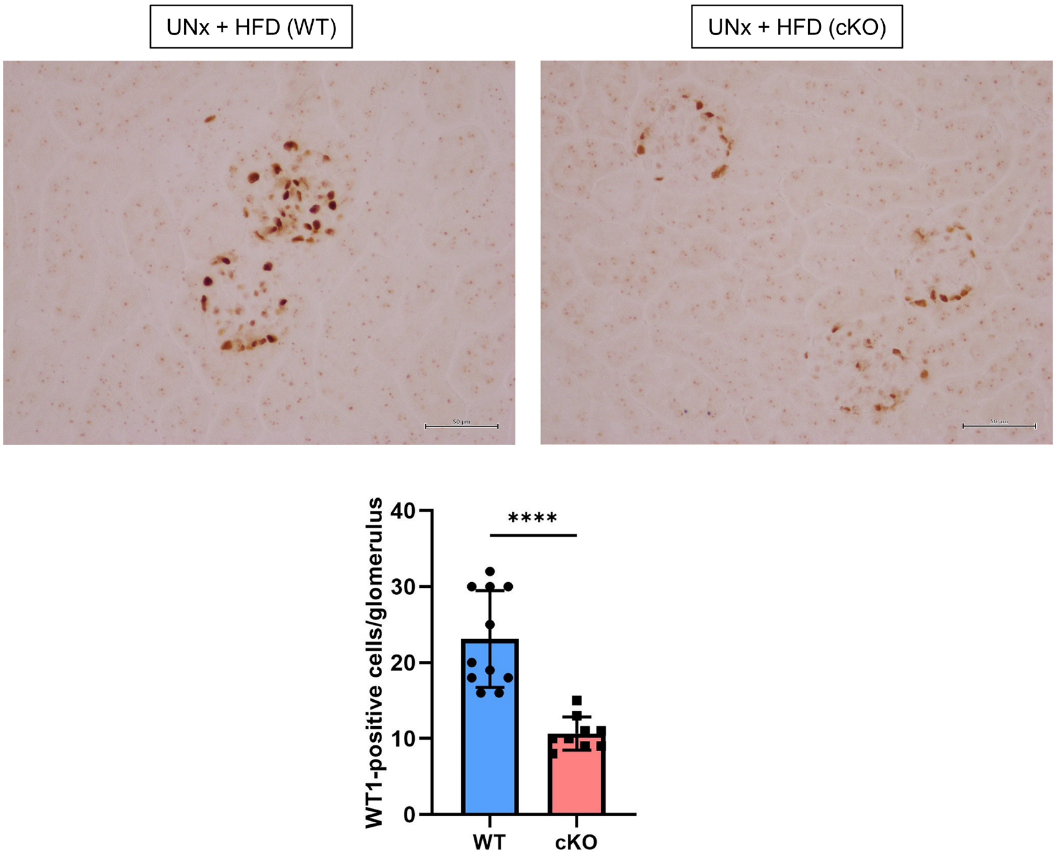

Supplementary Figure S4. ***Pdzd8*** **deletion reduces the number of podocytes per glomerulus in an accelerated obesity related kidney disease model**

The optical microscopic imaging with Wilms tumor 1 (WT1)-positive cells is shown. Scale bar = 50 μm. The number of WT1-positive cells per glomerulus is illustrated (n = 9 or 11). Data are presented as mean ± SD. *P*-value is determined by unpaired Student's *t* test. ****$P < 0.0001$.

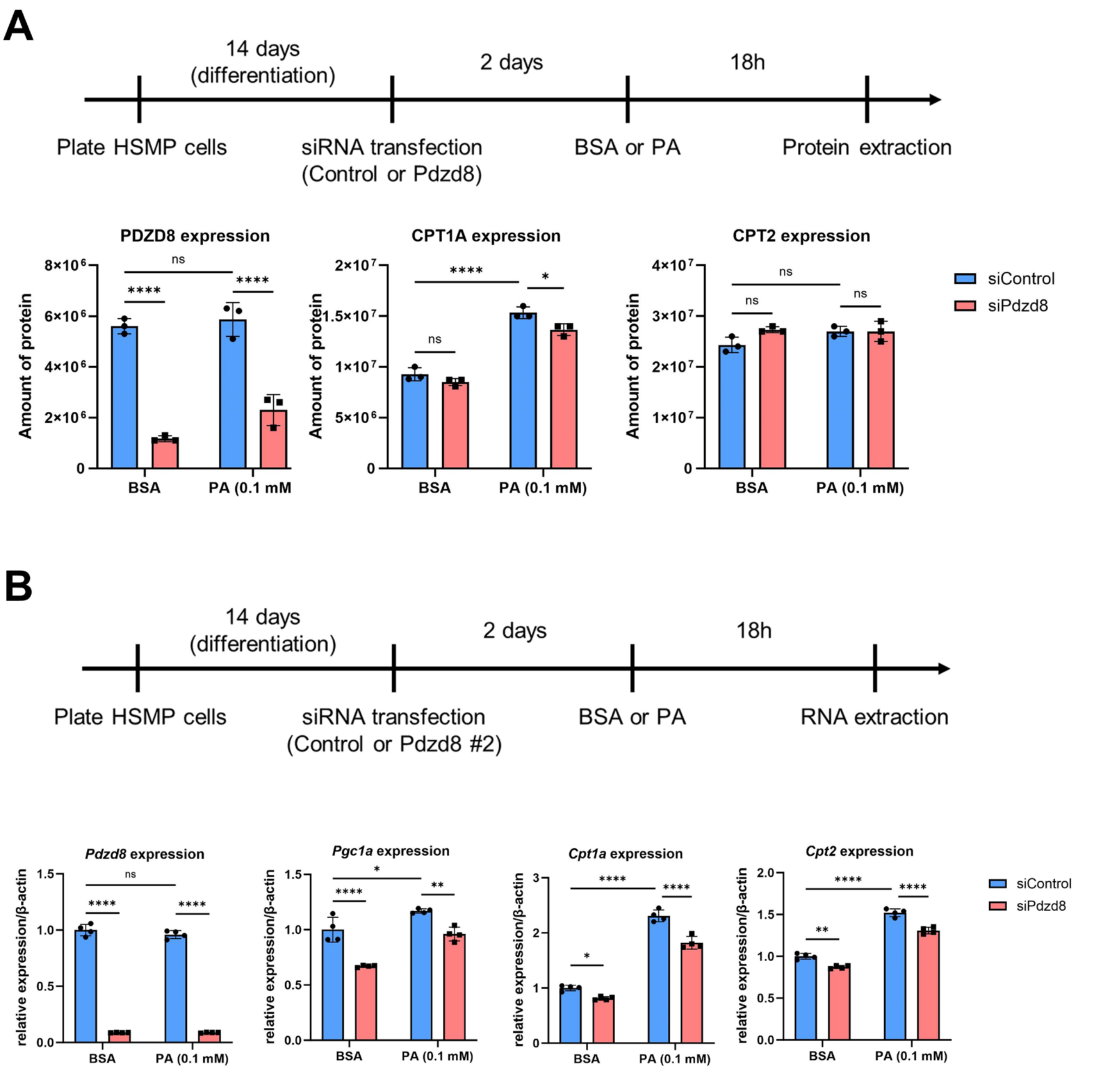

Supplementary Figure S5. *Pdzd8* knockdown inhibits the activity of mitochondria and fatty acid oxidation (FAO) in podocytes

(A) The results of proteome analysis are shown (n = 3, each).

(B) The experiments using the other *Pdzd8* siRNA (#2) are conducted. The results of quantitative real-time PCR are shown (n = 4, each).

HSMP, heat sensitive mouse podocytes; BSA, bovine serum albumin; PA, palmitic acids; *Pgc1a*, peroxisome proliferator-activated receptor gamma coactivator 1-alpha; *Cpt1a*, carnitine palmitoyltransferase-1a; *Cpt2*, carnitine palmitoyltransferase-2. Data are presented as mean ± SD. *P*-values are determined by 2-way ANOVA with Tukey's multiple-comparison test. *$P < 0.05$, **$P < 0.01$, ****$P < 0.0001$, ns, not significant.

A

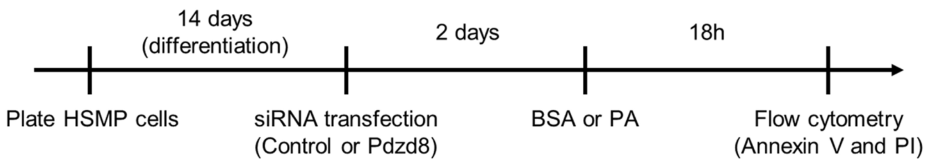

B

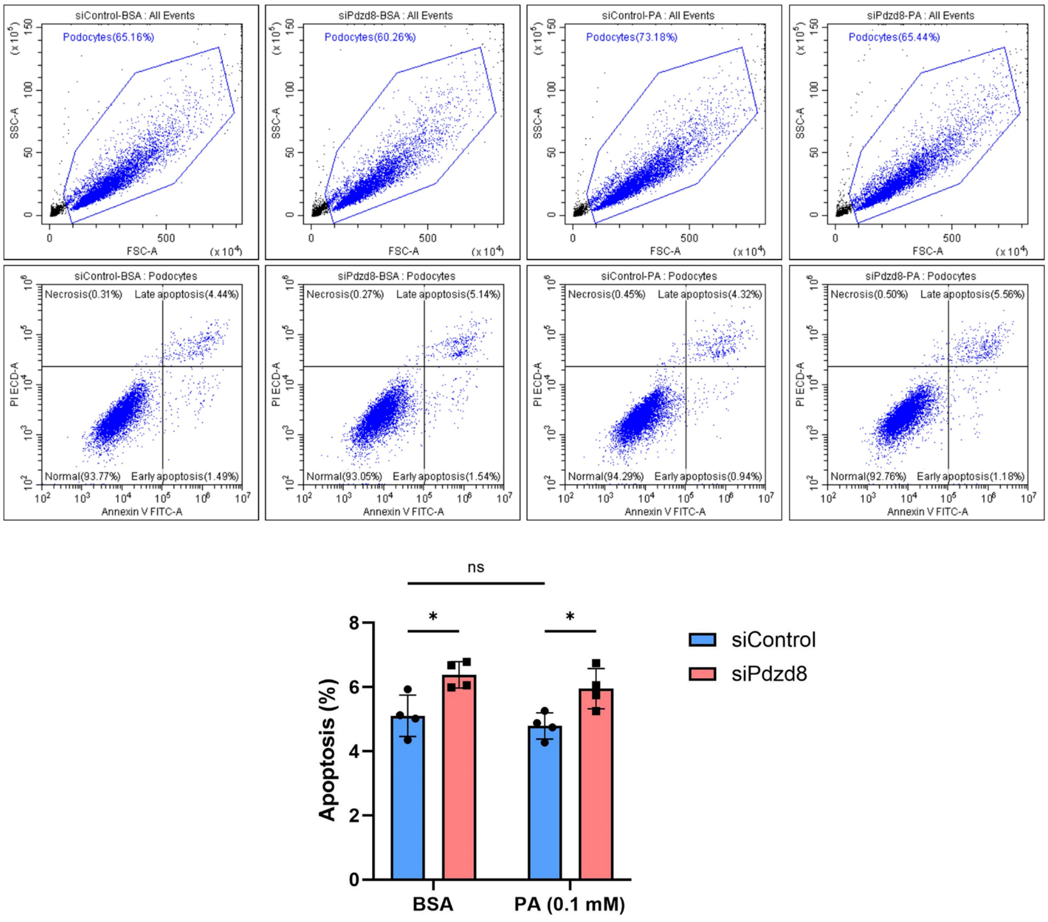

Supplementary Figure S6. *Pdzd8* knockdown increases the apoptosis of palmitic acids-treated podocytes

(A) The study protocol is shown.

(B) Flow cytometry analysis using Annexin V and propidium iodide (PI) is shown to assess the rate of podocyte apoptosis. The rate of apoptosis (early + late apoptosis) is illustrated (n = 4, for each). Data are presented as mean ± SD. *P*-values are determined by 2-way ANOVA with Tukey's multiple-comparison test. **P* < 0.05, ns, not significant.

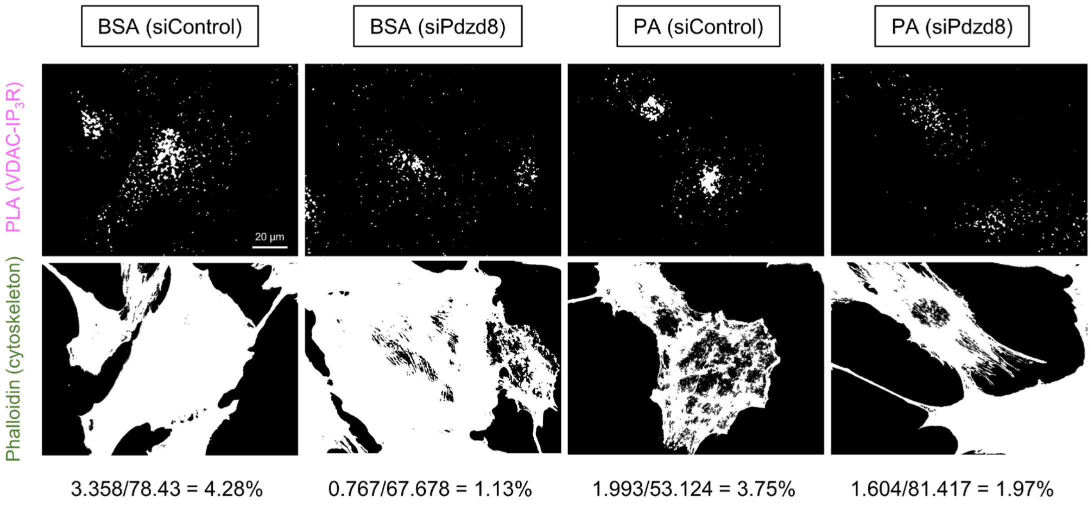

Supplementary Figure S7. Quantification of the ratio of proximity ligation signal to Phalloidin

The process of calculating the ratio of proximity ligation signal to Phalloidin in Figure 5 is shown. The appropriate thresholds for covering signal-positive areas are set by Image J software. The raw data are binary converted, according to each threshold. The signal-positive area is automatically calculated by macro-execution. Scale bar = 20 μm.

VDAC, Voltage dependent anion channel; IP_3R, Inositol 1,4,5-trisphosphate receptor.

A

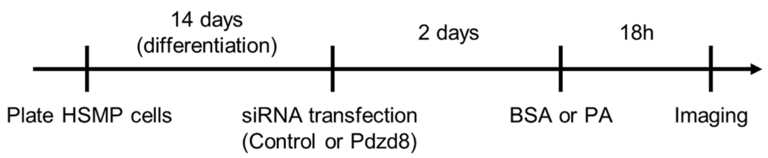

B

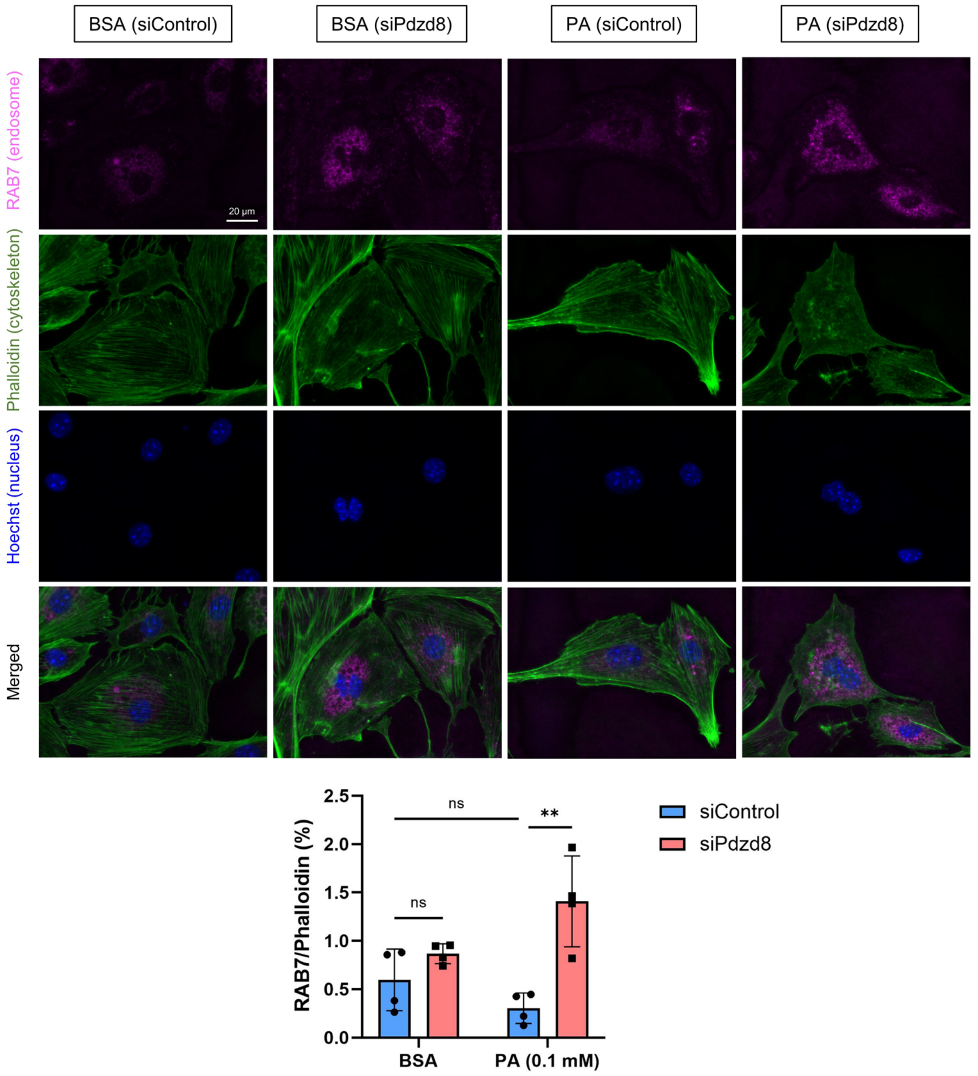

Supplementary Figure S8. Endosomes are accumulated in *Pdzd8* knockdown podocytes following palmitic acids treatment

(A) The study design is shown.

(B) RAB7 (endosomes), Phalloidin (cytoskeleton), Hoechst (nucleus) and merged pictures are shown. The process of calculating the ratio of RAB7 to Phalloidin is shown in Supplementary Figure S9 (n = 4, for each). Scale bar = 20 µm. Data are presented as mean ± SD. *P*-values are determined by 2-way ANOVA with Tukey's multiple-comparison test. ***P* < 0.01, ns, not significant.

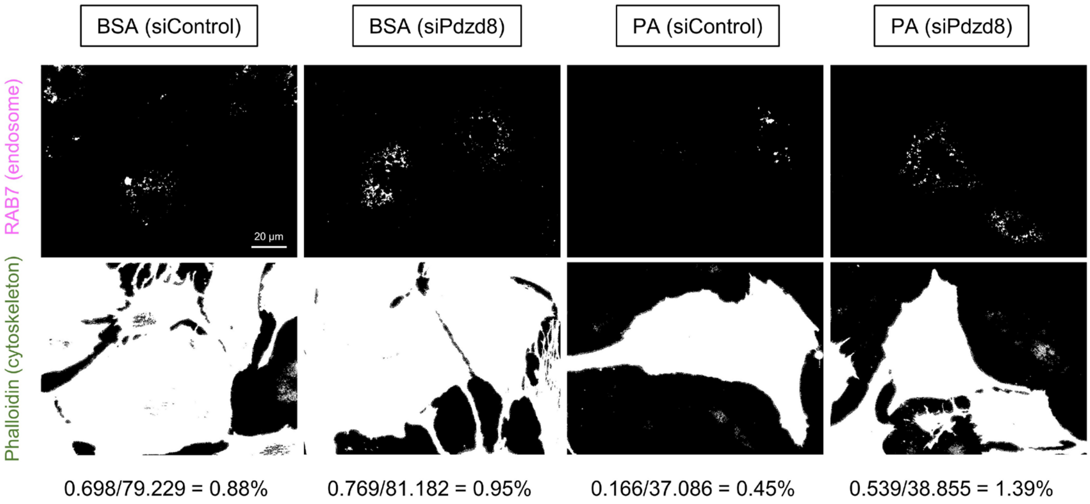

Supplementary Figure S9. Quantification of the ratio of RAB7 to Phalloidin
The process of calculating the ratio of RAB7 to Phalloidin in Supplementary Figure S8 is shown. The appropriate thresholds for covering signal-positive areas are set by Image J software. The raw data are binary converted, according to each threshold. The signal-positive area is automatically calculated by macro-execution. Scale bar = 20 μm.

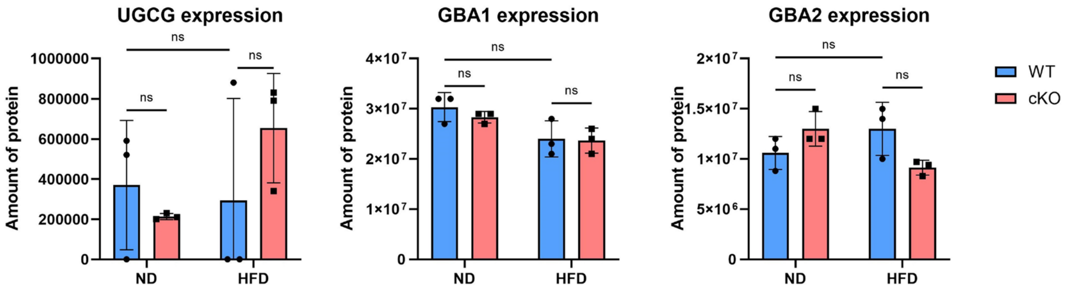

Supplementary Figure S10. *Pdzd8* knockout tends to increase UGCG expression and decrease GBA2 expression in the isolated glomeruli of a HFD model

The amount of UDP-glucose ceramide glucosyltransferase (UGCG), glucosylceramidase beta 1 (GBA1), and glucosylceramidase beta 2 (GBA2) proteins in the isolated glomeruli are shown (n = 3, for each). Data are presented as mean ± SD. *P*-values are determined by 2-way ANOVA with Tukey's multiple-comparison test. ns, not significant.

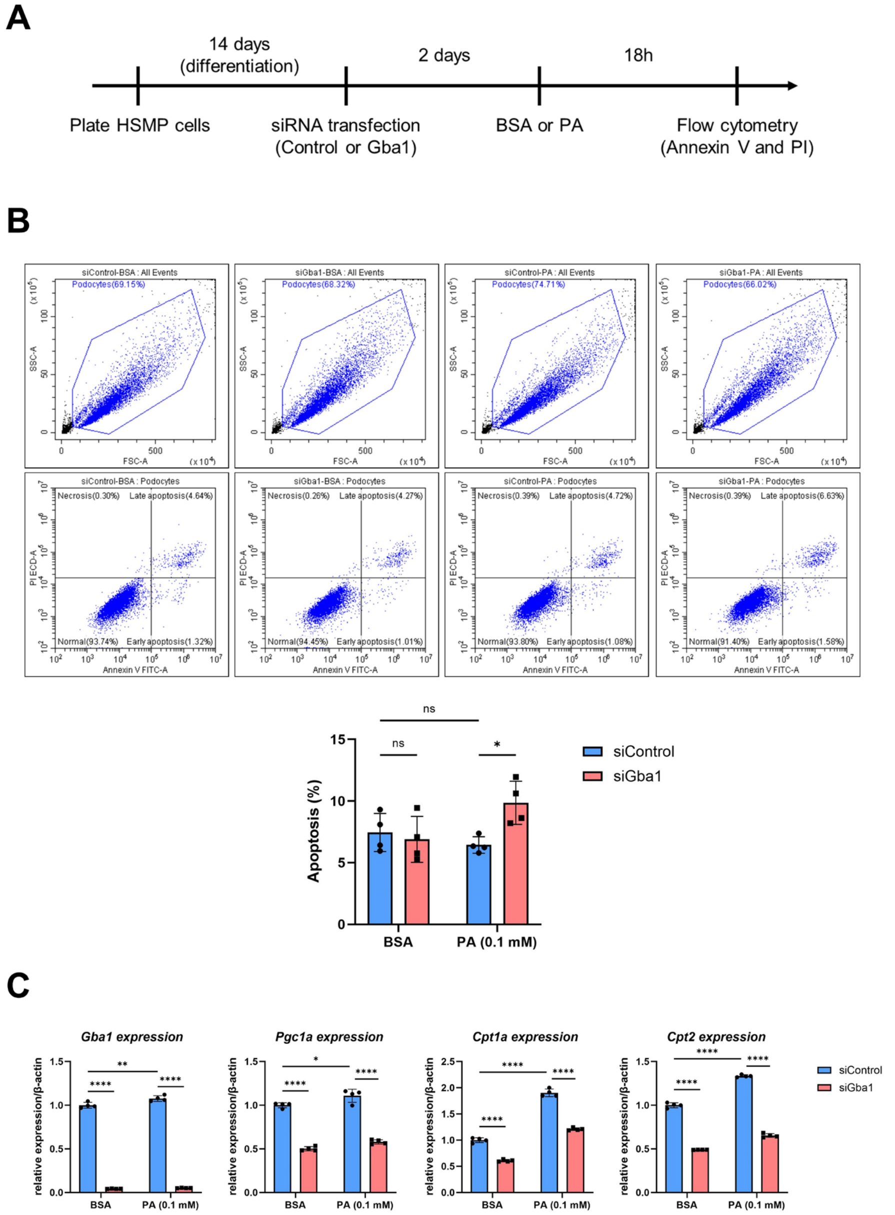

Supplementary Figure S11. *Gba1* knockdown increases the apoptosis of palmitic acids-treated podocytes, in association with the inhibition of mitochondrial activity

(A) The study protocol is shown.

(B) Flow cytometry analysis using Annexin V and propidium iodide (PI) is shown to assess the rate of podocyte apoptosis. The rate of apoptosis (early + late apoptosis) is illustrated (n = 4, for each). (C) The results of quantitative real-time PCR are shown (n = 4, each). *Gba1*, glucosylceramidase beta 1; *Pgc1a*, peroxisome proliferator-activated receptor gamma coactivator 1-alpha; *Cpt1a*, carnitine palmitoyltransferase-1a; *Cpt2*, carnitine palmitoyltransferase-2. Data are presented as mean ± SD. *P*-values are determined by 2-way ANOVA with Tukey's multiple-comparison test. *$P < 0.05$, **$P < 0.01$, ****$P < 0.0001$, ns, not significant.

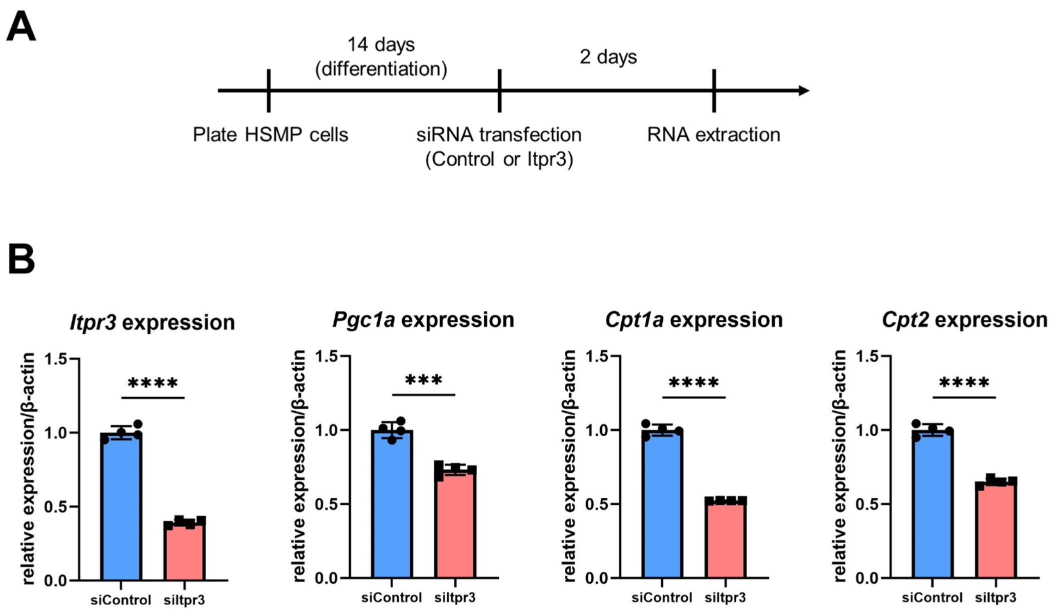

Supplementary Figure S12. The knockdown of *Itpr3*, another mitochondrial-ER contact sites factor, also induces mitochondrial inactivation in podocytes

(A) The study protocol is shown.

(B) The results of quantitative real-time PCR are shown (n = 4, each). *Itpr3*, Inositol 1,4,5-trisphosphate receptor type 3; *Pgc1a*, peroxisome proliferator-activated receptor gamma coactivator 1-alpha; *Cpt1a*, carnitine palmitoyltransferase-1a; *Cpt2*, carnitine palmitoyltransferase-2. Data are presented as mean ± SD. *P*-values are determined by unpaired Student's *t* test. ***$P < 0.001$, ****$P < 0.0001$.

(Supplementary Tables are provided as separate Excel files.)

Supplementary Table S1. The comprehensive proteome analysis of the mouse isolated glomeruli

ND (WT): ND11-13, ND (cKO): ND14-16, HFD (WT): HF11-13, HFD (cKO): HF14-16

Supplementary Table S2. The list of differentially expressed proteins between HFD-WT and HFD-cKO groups

Supplementary Table S3. The lipidome analysis of podocytes

Sheet 1: Results, Sheet 2: Hierarchical cluster analysis (HCA).

Supplementary Table S4. Primer sequences for the mouse genotyping (tail PCR)
